# Transcriptome-based screening in TARDBP/TDP-43 knock-in motor neurons identifies the NEDD8-activating enzyme inhibitor MLN4924

**DOI:** 10.1038/s41598-025-12147-8

**Published:** 2025-08-05

**Authors:** Sarah Lépine, Gilles Maussion, Alexandria Schneider, Angela Nauleau-Javaudin, María José Castellanos-Montiel, Georgina Jiménez Ambriz, Dan Spiegelman, Narges Abdian, Anna Krystina Franco-Flores, Ghazal Haghi, Lale Gursu, Michael R. Fiorini, Allison A. Dilliott, Sali M. K. Farhan, Mathilde Chaineau, Thomas M. Durcan

**Affiliations:** 1 Early Drug Discovery Unit (EDDU), The Neuro-Montreal Neurological Institute and Hospital, McGill University, Montreal, QC H3A 1A1 Canada; 2 Department of Neurology and Neurosurgery, McGill University, Montreal, QC H3A 1A1 Canada; 3 Faculty of Medicine and Health Sciences, McGill University, Montreal, QC H3G 2M1 Canada; 4 Faculté de médecine, Université de Montréal, Montreal, QC H3T 1J4 Canada; 5 The Neuro Bioinformatics Core Facility, The Neuro-Montreal Neurological Institute and Hospital, McGill University, Montreal, QC H3A 1A1 Canada; 6 Department of Human Genetics, McGill University, Montreal, QC H3A0G4 Canada

**Keywords:** Experimental models of disease, Motor neuron disease

## Abstract

A growing body of knowledge implicates perturbed RNA homeostasis in amyotrophic lateral sclerosis (ALS), a neurodegenerative disease that currently has no cure and few available treatments. Dysregulation of the multifunctional RNA-binding protein TDP-43 is increasingly regarded as a convergent feature of this disease, evidenced at the neuropathological level by the detection of TDP-43 pathology in most patient tissues, and at the genetic level by the identification of disease-associated mutations in its coding gene *TARDBP*. To characterize the transcriptional landscape induced by *TARDBP* mutations, we performed whole-transcriptome profiling of motor neurons (MNs) differentiated from two knock-in iPSC lines expressing the ALS-linked TDP-43 variants p.A382T or p.G348C. Our results show that the *TARDBP* mutations significantly altered the expression profiles of mRNAs and microRNAs of the 14q32 cluster in MNs. Using mutation-induced gene signatures and the Connectivity Map database, we identified compounds predicted to restore gene expression toward wild-type levels. Among top-scoring compounds selected for further investigation, the NEDD8-activating enzyme inhibitor MLN4924 effectively improved cell viability and neuronal activity, highlighting a possible role for protein post-translational modification via NEDDylation in the pathobiology of TDP-43 in ALS.

**Supplementary Information:**

The online version contains supplementary material available at 10.1038/s41598-025-12147-8.

## Introduction

Amyotrophic lateral sclerosis (ALS) is a neurodegenerative disorder caused by the progressive degeneration of motor neurons (MNs) in the brain and the spinal cord resulting in weakness, loss of ambulation, and eventual fatal paralysis of respiratory function^1^. From disease onset, the mean survival ranges from two to four years^2^. At present, the few available treatments (i.e., riluzole^3–5^, edaravone^6,7^) offer modest benefits or are only efficacious in a subset of patients, as is the case for the SOD1-lowering therapy tofersen^8–10^. Limitations in treating these patients reflect our incomplete understanding of the molecular basis of ALS and the difficulty in therapeutically addressing the multifactorial nature of the disease.

Several lines of evidence implicate disturbed RNA metabolism in ALS. A pathological hallmark of ALS is the nuclear depletion and cytoplasmic aggregation of TAR DNA-binding protein 43 (TDP-43), an RNA-binding protein encoded by *TARDBP* involved in nearly all aspects of RNA metabolism^11–13^. Mutations in *TARDBP* and several other genes encoding RNA-binding proteins (e.g., *FUS*, *HNRNPA1*, *HNRNPB2*, *MATR3*,* TAF15*) have been implicated in ALS^14,15^. Furthermore, transcriptome alterations have been repeatedly reported in the brain and spinal cord of patients who have succumbed to this disease^16–23^, establishing a strong link between perturbed RNA homeostasis and ALS.

Initially identified as a transcriptional repressor^24^, TDP-43 has since been implicated in splicing^25^, microRNA (miRNA) biogenesis^26,27^, RNA stability and transport^28,29^, and translation^30^. In keeping with these functions, TDP-43 localizes to ribonucleoprotein (RNP) condensates both in the nucleus (i.e., nuclear speckles^31^, paraspleckles^32^, and Cajal bodies^32^) and the cytoplasm (i.e., transport granules^28,29^, stress granules^33–35^, and processing (P)-bodies^30^), which broadly serve to regulate gene expression in time and space. While the many roles of TDP-43 in RNA metabolism have been extensively documented, the impact of disease-associated mutations in *TARDBP* on the RNA landscape is still being determined. Several studies have reported RNA abnormalities in *TARDBP*/*Tardbp* mutant mouse models^36–41^, but whether these observed changes accurately reflect the human condition has been questioned.

As dysregulation of gene expression can have broad downstream consequences, determining the transcriptome alterations that arise in ALS can inform the development of transcriptome-correcting therapies. This drug discovery paradigm, known as “transcriptome reversal”^42 ^could give rise to therapies able to normalize several disease pathways simultaneously.

To this end, we performed whole-transcriptome profiling of MNs differentiated from two knock-in induced pluripotent stem cell (iPSC) lines expressing the ALS-linked TDP-43 variants p.A382T or p.G348C, leading to the identification of mutation-induced RNA signatures. Positing that shared alterations may be reflective of underlying disease mechanisms, we queried the Connectivity Map (CMap) database^43^ for compounds predicted to normalize gene expression changes toward wild-type levels. Selected top-scoring compounds identified *in silico* were tested experimentally for their ability to ameliorate previously identified disease-relevant readouts^44^ in two phenotypic screens for viability and neuronal activity. This approach led to the identification of the NEDD8-activating enzyme inhibitor MLN4924.

## Results

### Transcriptomic profiling of MNs differentiated from *TARDBP* knock-in iPSCs

**Fig. 1:**
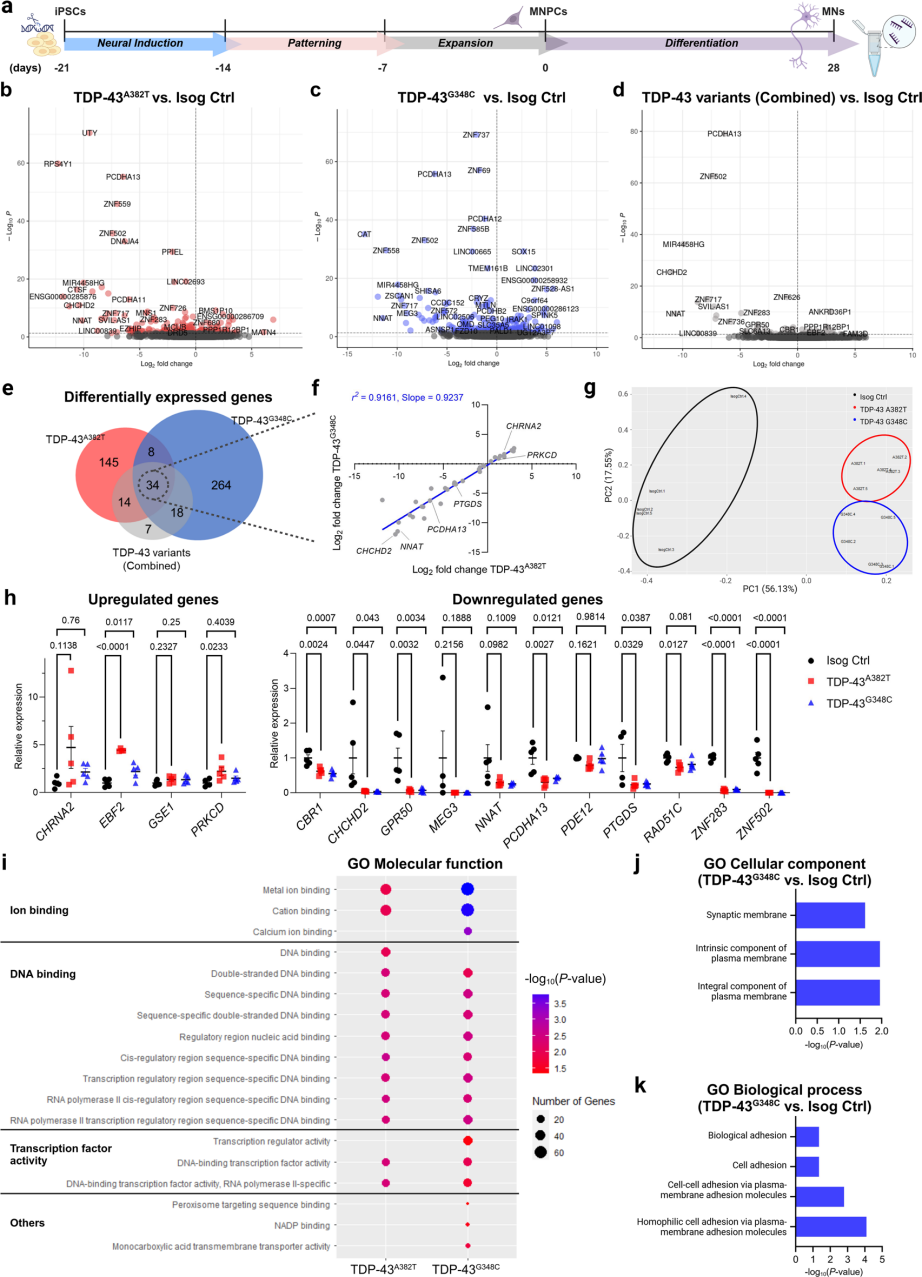
Differential gene expression analysis in TDP-43^A382T^ and TDP-43^G348C^ MNs. (**a**) Schematic representation of iPSCs differentiation into MN progenitor cells (MNPCs) and MNs for total RNA extraction. (**b**–**e**) Volcano plots (**b**–**d**) and Venn diagram (**e**) comparing differentially expressed genes (DEGs) in TDP-43 MNs differentiated for 28 days (4 weeks) relative to isogenic control. *n* = 5 independent differentiations. (**f**) Scatter plot showing a strong correlation between fold changes of overlapping DEGs. (**g**) Principal component analysis (PCA)-based clustering of RNA-seq samples using Log_10_(*n* + 1) transformed normalized read counts of DEGs from the combined contrast. Individual points represent biological replicates from independent differentiations. (**h**) Validation of selected common DEGs by qPCR. Data shown as mean ± SEM. One-way ANOVA with Dunnett’s post hoc test. *P*-values shown. Significance was defined as *P* < 0.05. *n* = 5 independent differentiations. (**i**) Representative gene ontology (GO) terms of the “molecular function” category enriched in DEGs of TDP-43^A382T^ and TDP-43^G348C^ MNs. (**j**,**k**) Representative GO terms of the “cellular component” (**j**) and “biological process” (**k**) categories enriched in DEGs of TDP-43^G348C^ MNs. Of note, genes dysregulated in TDP-43^A382T^ MNs were not significantly enriched in GO terms of these categories.

We have recently reported the generation of two homozygous knock-in iPSC lines with mutations in *TARDBP* coding for TDP-43^A382T^ or TDP-43^G348C^, two frequent ALS variants of TDP-43^44^. To characterize the transcriptomic profiles of MNs differentiated from those cells, we performed next-generation RNA sequencing (RNA-seq) on total RNA extracted from mutant and isogenic control iPSC-derived MN cultures at 4-weeks post-plating of MN progenitor cells (MNPCs) (Fig. 1a, Supplementary Table S1). Analysis of normalized counts confirmed the expression of the MN markers *ISL1*, *MNX1* (Hb9), *CHAT*, and *SLC18A3* (VAChT) (Supplementary Fig. S1a). Unsurprisingly, *MNX1* (Hb9) was detected at relatively low levels as this early-MN marker is gradually downregulated with maturation in limb-innervating MNs^45^. In addition, we noted the expression of the V2 interneuron markers *LMO4*, *VSX2*/*CHX10*, and *SOX14*, reflecting the anatomical proximity of V2 and pMN domains in the developing spinal cord. Markers for astrocytes, oligodendrocytes, neuronal progenitors, and V1/V3 interneurons were mostly absent or expressed at low levels (Supplementary Fig. S1a). Expression of the stem cell marker *NANOG* was not detected and *POU5F1* (Oct-4) levels were negligible. These results suggest a mixed population of cells enriched in ventral spinal neurons with the presence of some glial and progenitor cells, consistent with previous single-cell RNA-seq experiments^46^. Importantly, the expression levels of cell type markers did not significantly differ between mutant and control samples, indicating a similar cell composition and differentiation propensity, as previously described^44^.

### TDP-43^A382T^ and TDP-43^G348C^ affect similar genes and pathways in human MNs

To identify transcriptional changes induced by *TARDBP* mutations, we performed differential gene expression analysis using DESeq2 (false discovery rate [FDR] < 0.05) by contrasting each mutation to the isogenic control (Fig. 1b-c and Supplementary Data S1). We identified 201 differentially expressed genes (DEGs) in TDP-43^A382T^ MNs compared with control (137 downregulated genes and 64 upregulated genes), and 324 DEGs in TDP-43^G348C^ MNs compared with control (161 downregulated genes and 163 upregulated genes) (Supplementary Fig. S1c-d). The expression levels of the ALS-linked genes *SOD1*, *C9ORF72*, *FUS*, and *TARDBP* did not differ between TDP-43 and isogenic control samples (Supplementary Fig. S1b). When comparing the genes dysregulated in TDP-43^A382T^ and TDP-43^G348C^ MNs, there was a significant overlap (*P* < 1.004e-41, hypergeometric test), revealing a total of 42 DEGs in common (Supplementary Fig. S1d, Supplementary Table S3). Additionally, we combined the datasets from the two mutations and performed differential expression analysis against the isogenic control (TDP-43^A382T^ + TDP-43^G348C^ vs. Isog Ctrl) (Fig. 1d, Supplementary Data S1), with the goal of identifying DEGs with a shared direction of effect in TDP-43 samples. This analysis yielded a total of 73 DEGs. Among those, 14 and 18 genes were uniquely dysregulated in TDP-43^A382T^ and TDP-43^G348C^ MNs respectively in the prior analyses, while 34 genes were shared between the individual and combined contrasts (Fig. 1e). There was a strong correlation between fold changes in shared differentially expressed genes (*r*^*2*^ = 0.9161, slope = 0.9237, *P* < 0.0001) (Fig. 1f), indicating a similar magnitude of effect on gene expression by the two mutations. The convergence between TDP-43^A382T^ and TDP-43^G348C^ MNs was corroborated by findings from a principal-component analysis of DEGs (Fig. 1g), where PC2 distinguished TDP-43^A382T^ and TDP-43^G348C^ samples into two distinct but closely related clusters.

We selected a subset of genes for validation by qPCR. We focused on genes showing an overlap between TDP-43^A382T^ and TDP-43^G348C^ MNs, particularly those related to cellular processes relevant to ALS and/or associated with ALS or more broadly with neurological disorders (Supplementary Table S3). We also took into consideration the expression levels (i.e., normalized counts) and the log fold changes between conditions, reasoning that a greater magnitude of change and/or alterations in the levels of highly expressed genes are more likely to result in biologically significant effects. These strategies facilitated the selection of a total of 15 genes among those dysregulated in TDP-43 MNs. Differential expression of 10 genes was confirmed in either or both *TARDBP* mutants in five additional RNA samples from a separate set of extractions (*EBF2*, *PRKCD*, *CRB1*, *CHCHD2*, *GPR50*, *PCDHA13*, *PTGDS*, *RAD51C*, *ZNF283*, *ZNF502*) (Fig. 1h).

In addition to a shared gene signature, Gene Ontology (GO) enrichment analyses revealed that both mutants showed a dysregulation in genes with molecular functions related to ion binding, DNA binding, and transcription factor activity (Fig. 1i), consistent with altered DNA/RNA-binding protein function. Moreover, genes dysregulated in TDP-43^G348C^ MNs showed a significant enrichment in the GO cellular component terms “Integral component of plasma membrane”, “intrinsic component of plasma membrane”, and “synaptic membrane” as well as in GO biological processes related to cell adhesion (Fig. 1j, k). Interestingly, the “cell adhesion” category has similarly been reported to be enriched in several transcriptomic studies of ALS patient samples and iPSC-derived MN models^21,47,48^. Genes dysregulated in TDP-43^A382T^ MNs were not significantly enriched in GO terms of the “cellular component” and “biological processes” categories.

**Fig. 2:**
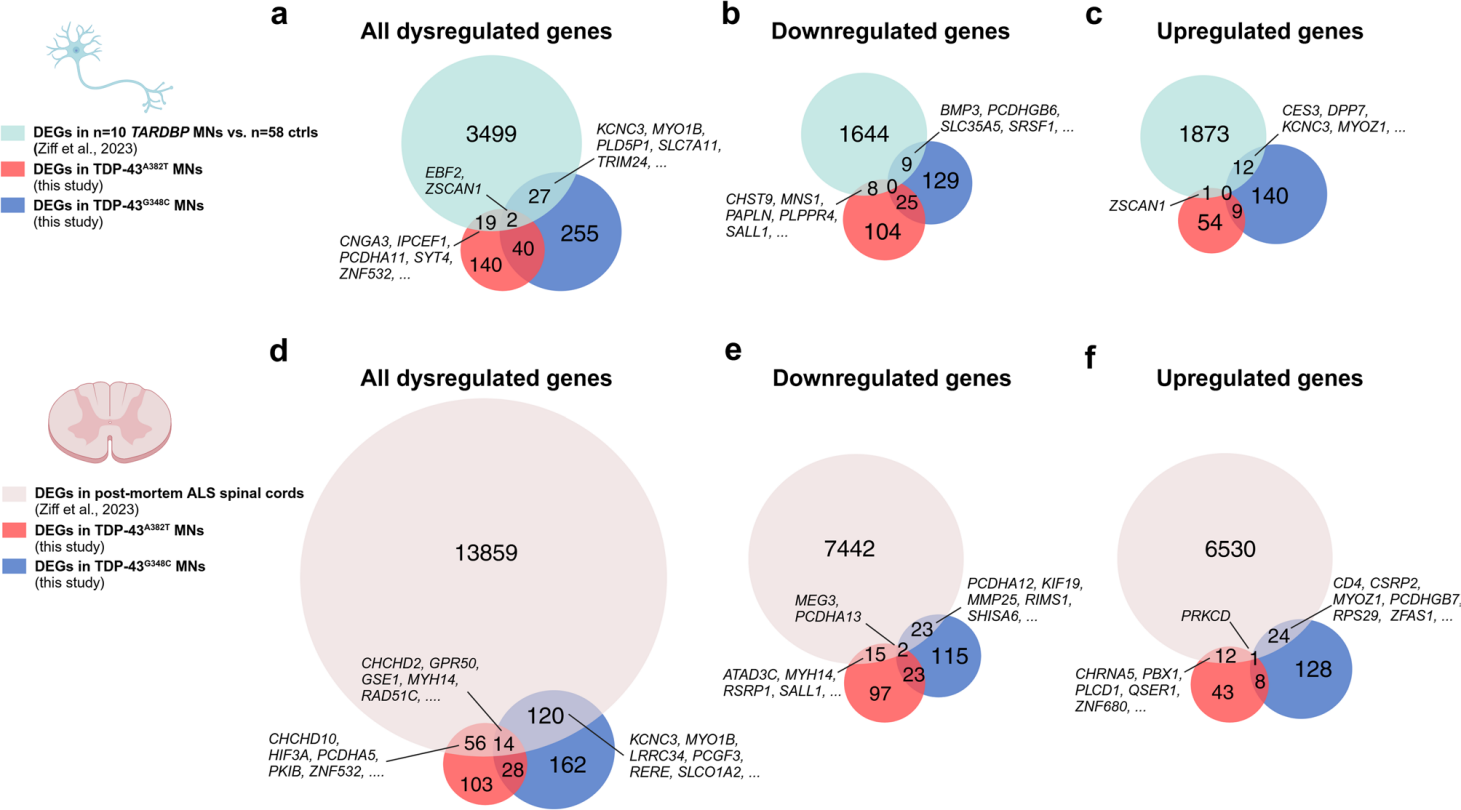
Overlapping RNA alterations across datasets from *TARDBP* mutant iPSC-derived MNs and post-mortem ALS spinal cords. (**a**–**c**) Venn diagram comparing differentially expressed genes (DEGs) in TDP-43^A382T^ and TDP-43^G348C^ MNs with other *TARDBP* mutant iPSC-derived MNs (*n* = 10 *TARDBP* MN lines vs. *n* = 58 controls)^49^. (**d**–**f**) Venn diagram comparing DEGs in TDP-43^A382T^ and TDP-43^G348C^ MNs with post-mortem spinal cords from patients with genetic and non-genetic forms of ALS (*n* = 214 post-mortem ALS spinal cords [*n* = 161 non-genetic ALS, *n* = 36 C9orf72, *n* = 5 SOD1, *n* = 2 FUS, and *n* = 10 with mutations in 8 other ALS genes] vs. *n* = 57 control specimens)^49^.

To determine whether the gene expression changes identified in our TDP-43 models resemble those dysregulated in other *TARDBP* mutant iPSC-derived MNs, we turned to a database by Ziff and colleagues^49^ (available from https://oliverziff.shinyapps.io/als_genome_instability/) which catalogs differential expression data generated from RNA-seq datasets of 429 ALS and control iPSC-derived MN lines. Focusing on *TARDBP* mutations (*n* = 58 controls vs. *n* = 10 *TARDBP* lines [p.Q331K, p.M337V, p.I383T, and p.N390D], we retrieved a total of 3547 DEGs, with 1661 downregulated and 1886 upregulated genes. Comparing this gene set with ours, we found a modest but statistically significant overlap (TDP-43^A382T^: *P* < 2.802e-06, TDP-43^G348C^: *P* < 1.798-11, hypergeometric test) (Fig. 2a, Supplementary Data S2). Approximately 10.4% (21/201) of DEGs in TDP-43^A382T^ MNs and 9.0% (29/324) of DEGs in TDP-43^G348C^ MNs were also found to be dysregulated in the retrieved *TARDBP* mutant dataset. When narrowing our search to genes with a concordant directionality of change, we found 8 co-downregulated genes and 1 co-upregulated between TDP-43^A382T^ MNs and the compared *TARDBP* dataset (Fig. 2b, c). Among genes dysregulated in TDP-43^G348C^ MNs, 9 and 12 genes were co-downregulated and co-upregulated in the *TARDBP* dataset, respectively (Fig. 2b, c). Furthermore, similar to our findings, the *TARDBP* MNs showed a significant enrichment in GO terms related to RNA processing and synaptic function^49^. Additional functional terms included “protein binding”, “microtubule cytoskeleton”, and “nervous system development”. Thus, though distinct variants were contrasted in the dataset from Ziff and colleagues, it appears that *TARDBP* mutations converge, to some extent, on similar genes and pathways.

### TDP-43 MNs share gene expression changes with post-mortem ALS spinal MNs

To further assess the relevance of our findings, we compared our data with a previously published dataset obtained from post-mortem spinal cord specimens from 57 neurologically healthy individuals and 214 ALS patients from the NYGC ALS cohort (*n* = 161 non-genetic ALS, *n* = 36 *C9orf72*, *n* = 5 *SOD1*, *n* = 2 *FUS*, and *n* = 10 with mutations in 8 other ALS genes), available on the database by Ziff and colleagues^49^ queried above. We retrieved a total of 14,049 DEGs in ALS samples compared with controls (FDR < 0.05); specifically, 6567 upregulated genes and 7482 downregulated genes. When comparing all dysregulated genes, 34.8% (70/201) and 41.4% (134/324) of genes dysregulated in TDP-43^A382T^ and TDP-43^G348C^ MNs, respectively, were shared with post-mortem MNs (TDP-43^A382T^: *P* < 6.457e-129, TDP-43^G348C^: *P* < 2.338e-13, hypergeometric test) (Fig. 2d, Supplementary Data S2). A total of 14 genes were commonly dysregulated across all contrasts. When considering directionality of change, 17 downregulated genes and 15 upregulated genes were common between TDP-43^A382T^ MNs and post-mortem ALS spinal cords (Fig. 2e, f). A total of 25 downregulated genes and 25 upregulated genes were shared between post-mortem and TDP-43^G348C^ MNs (Fig. 2e, f). Among those, three genes were shared between both mutants and post-mortem samples (*PCDHA13* and *MEG3*, downregulated; *PRKCD*, upregulated) (Fig. 2e, f). Thus, *TARDBP* mutant iPSC-derived MNs recapitulate some of the transcriptional dysregulations observed in post-mortem ALS spinal cord samples.

### Few dysregulated genes are known TDP-43 targets

To further investigate the mechanisms underlying RNA changes induced by *TARDBP* mutations, we next considered a relationship between differential gene expression and mRNA binding by TDP-43. We turned to a previous study characterizing TDP-43 target mRNAs by enhanced crosslinking and immunoprecipitation (eCLIP) followed by RNA-seq in motor cortex specimens from sporadic ALS patients and neurologically healthy individuals^19^. We compared our DEGs with a list of genes whose mRNAs were previously reported to be bound by TDP-43 (Supplementary Fig. S2). We didn’t find a significant enrichment for TDP-43 targets within our datasets (TDP-43^A382T^ *P* < 0.086; TDP-43^G348C^ *P* < 0.177, hypergeometric test). Only a small fraction of DEGs were known TDP-43 targets (~ 2.5% in TDP-43^A382T^ and ~ 1.8% in TDP-43^G348C^). Two TDP-43-bound genes were dysregulated in both TDP-43 MN lines (*PTGDS*, *MEG3*), while three and four TDP-43-bound genes were only dysregulated in TDP-43^A382T^ (*ZNF532*, *PAPLN*, *TNS1*) or TDP-43^G348C^ MNs (*GRIN1*, *ARF3*, *CNTNAP4*, *RERE*), respectively. Among dysregulated TDP-43-bound genes, the majority (6/9) were downregulated. Given that, to our knowledge, similar cross-linking experiments characterizing TDP-43-bound transcripts have not yet been performed in human spinal cord specimens or spinal MN cultures, additional RNA-protein interaction studies are needed to reveal cell type-specific TDP-43 binding targets. In line with this idea, recent studies have shown that different cellular environments can modulate the RNA processing functions of TDP-43^50,51^.

### MicroRNA biogenesis is altered in mutant MNs

**Fig. 3:**
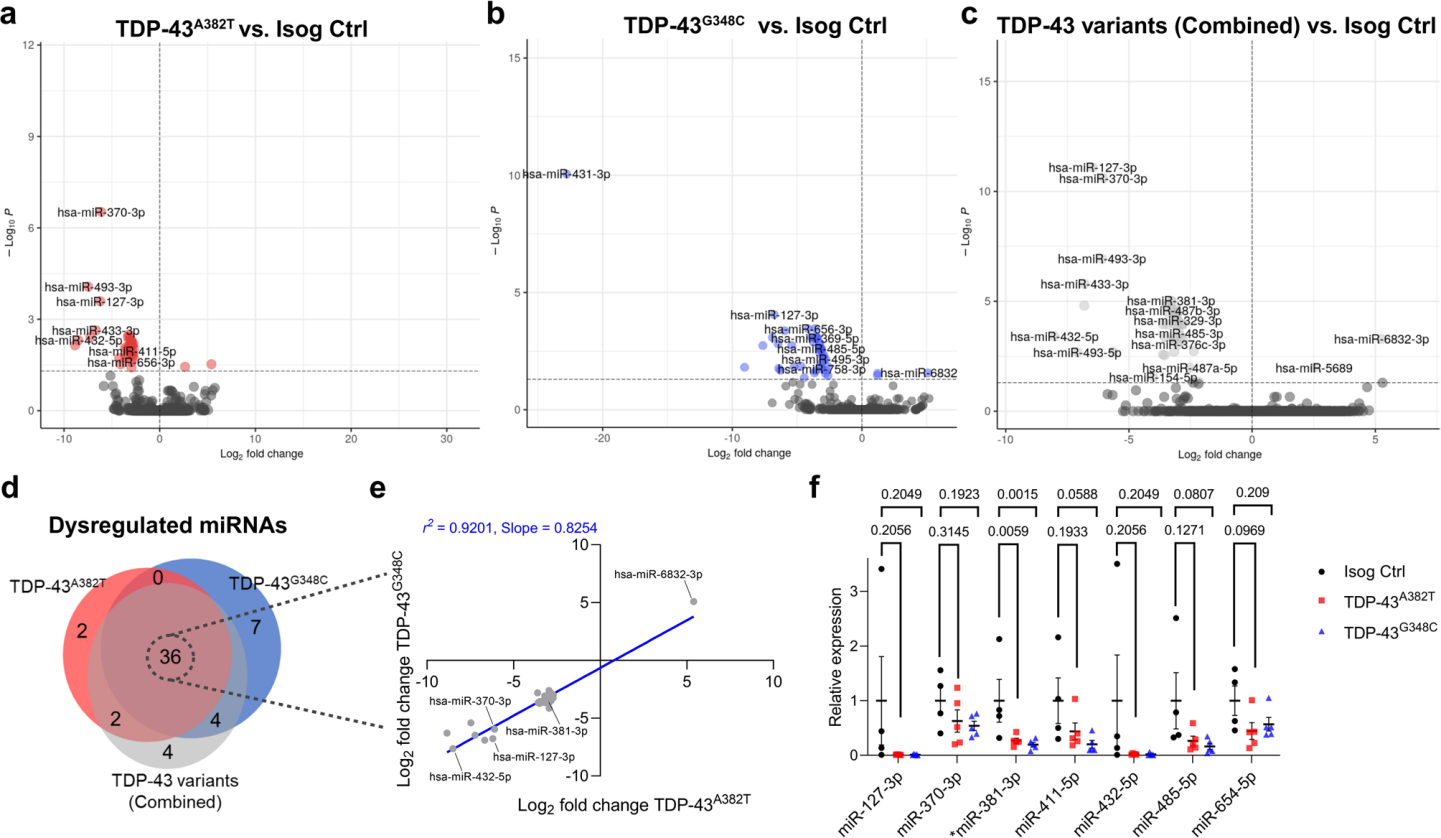
Differential miRNA expression analysis in mutant MNs. (**a**–**d**) Volcano plots (**a**–**c**) and Venn diagram (**d**) comparing differentially expressed miRNAs in TDP-43 MNs relative to isogenic control. *n* = 5 independent differentiations. (**e**) Scatter plot showing a strong correlation between fold changes of overlapping dysregulated miRNAs. (**f**) Validation of selected common dysregulated miRNAs by qPCR. Data shown as mean ± SEM. One-way ANOVA with Dunnett’s post hoc test. *P*-values shown. Significance was defined as *P < 0.05. *n* = 5 independent differentiations.

Given the roles of TDP-43 in miRNA biogenesis^27,52^, we next interrogated whether *TARDBP* mutations could indirectly affect gene expression through changes in miRNA abundance. To this end, we performed miRNA profiling by small RNA sequencing to characterize miRNA abundance in TDP-43^A382T^ and TDP-43^G348C^ MN cultures. We conducted differential expression analyses (FDR < 0.05) using the same contrast design used in the RNA-seq experiments (Fig. 3a-c and Supplementary Data S3). Differential expression analyses revealed a total of 40 and 47 miRNAs with an altered abundance in TDP-43^A382T^ and TDP-43^G348C^ MNs, respectively. The two mutants had largely overlapping dysregulated miRNAs (*P* < 0.05, hypergeometric test), with 36 miRNAs in common (Supplementary Fig. S3a, Supplementary Table S4). The combined analysis (TDP-43^A382T^ + TDP-43^G348C^ vs. Isog Ctrl) identified a total of 46 dysregulated miRNAs, including the 36 miRNAs shared with the individual analyses (Fig. 3d). Most miRNAs showed decreased abundance with a strong correlation in fold changes between the two mutants (*r*^*2*^ = 0.9201, slope = 0.8254, *P* < 0.0001) (Fig. 3e), which could indicate altered miRNA biogenesis or excess degradation. Downregulation was confirmed in 1 out of 7 miRNAs selected for validation (has-miR-381-3p), although most miRNAs showed a clear trend toward decreased abundance relative to the isogenic control (Fig. 3f).

Given that TDP-43 can directly associate with pri-miRNA and pre-miRNA precursors during miRNA maturation^27,52^, we queried the RBPmap database^53^ to search for TDP-43 binding motifs in the transcribed sequences of dysregulated miRNAs. We found one or more predicted TDP-43 binding sites in nearly half of the shared dysregulated miRNAs (17/36 miRNAs, Supplementary Table S5). Interestingly, we also noticed that the genomic coordinates of all 35 downregulated miRNAs mapped to the 14q32 miRNA cluster (Supplementary Table S4), which strongly implies that their expression is co-regulated. Accordingly, most of them were downregulated by the same magnitude (~ 3 log fold-changes) in TDP-43 samples relative to the isogenic control in differential expression analyses (Fig. 3e, Supplementary Table S4). Taken together, these results indicate that mutations in *TARDBP* coding for TDP-43^A382T^ and TDP-43^G348C^ lead to a decreased abundance of 14q32-encoded miRNAs.

### Functional consequences of dysregulated miRNA in mutant MNs

**Fig. 4:**
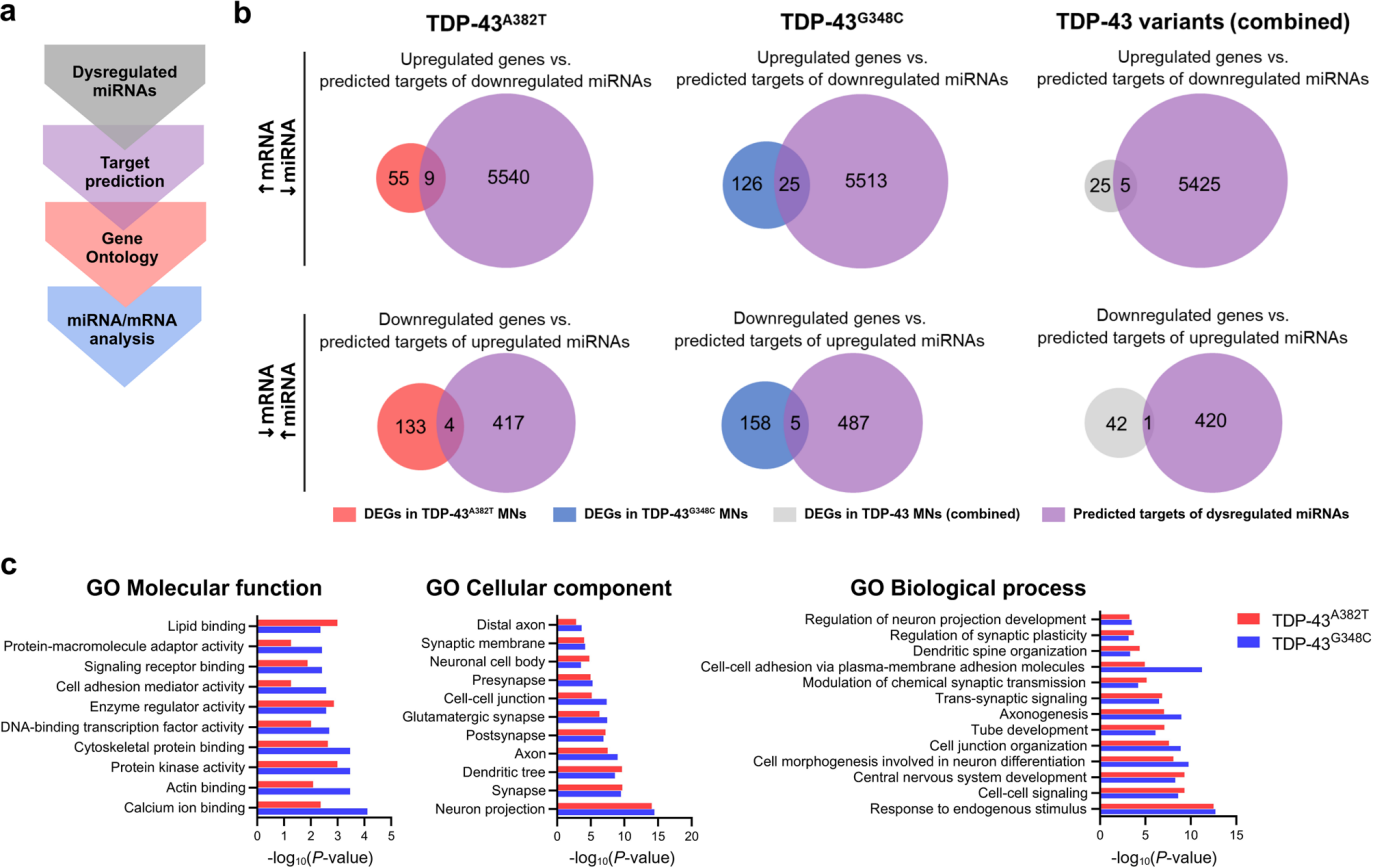
Functional characterization of dysregulated miRNAs. (**a**) Bioinformatics analysis workflow for functional characterization of dysregulated miRNAs. (**b**) Integrated mRNA/miRNA analysis comparing DEGs and predicted mRNA targets of dysregulated miRNAs in TDP-43 MNs. (**c**) Representative common enriched GO terms of predicted mRNA targets of dysregulated miRNAs.

To explore the potential functional consequences of miRNA perturbances, we used miRgate^54^ to predict targets of shared dysregulated miRNAs (Fig. 4a, Supplementary Data S4), filtering for target genes predicted by at least two out of five computational algorithms used by this database. Given that miRNAs can regulate gene expression post-transcriptionally through mRNA degradation, we considered an inverse correlation between dysregulated miRNAs and the expression levels of their predicted target mRNAs. The list of predicted genes targeted by downregulated miRNAs was compared to genes upregulated in mutant MNs determined by RNA-seq data (and vice versa). These mRNA/miRNA cross-analyses yielded a total of 13 and 30 genes identified as inversely correlated to miRNA abundance in TDP-43^A382T^ and TDP-43^G348C^ MNs, respectively, while a total of 6 genes were identified in the combined analysis (TDP-43^A382T^ + TDP-43^G348C^ vs. Isog Ctrl) (Fig. 4b, Supplementary Fig. S3b). These results indicate that the detected changes in gene expression could be partially explained by impaired miRNA biogenesis. Interestingly, we noted that several negatively correlated genes encode protocadherins (Supplementary Fig. S3b), which are cell-cell adhesion proteins enriched in the central nervous system (CNS)^55^. Upon re-examination of the RNA-seq data, we also found that DEGs were statistically enriched for the clustered protocadherin locus on chromosome 5 in both mutants (FDR < 0.05) (Supplementary Fig. S3d).

Aside from targeted mRNA degradation, miRNAs can achieve RNA silencing through translational repression, with little to no influence on mRNA target abundance^56^. To gain further insights into the pathways regulated by altered miRNA, we performed GO enrichment analyses of predicted target genes and investigated shared enriched GO terms per category. Overall, there was a common enrichment in GO terms related to synaptic function, cell adhesion, and neuronal development (Fig. 4c). Furthermore, KEGG pathway analysis^57,58^ identified a common enrichment in pathways related to cell signaling, axon guidance, and neurodegeneration (Supplementary Fig. S3c).

### *In silico* screen identifies compounds predicted to restore mutation-induced gene expression signatures

**Fig. 5:**
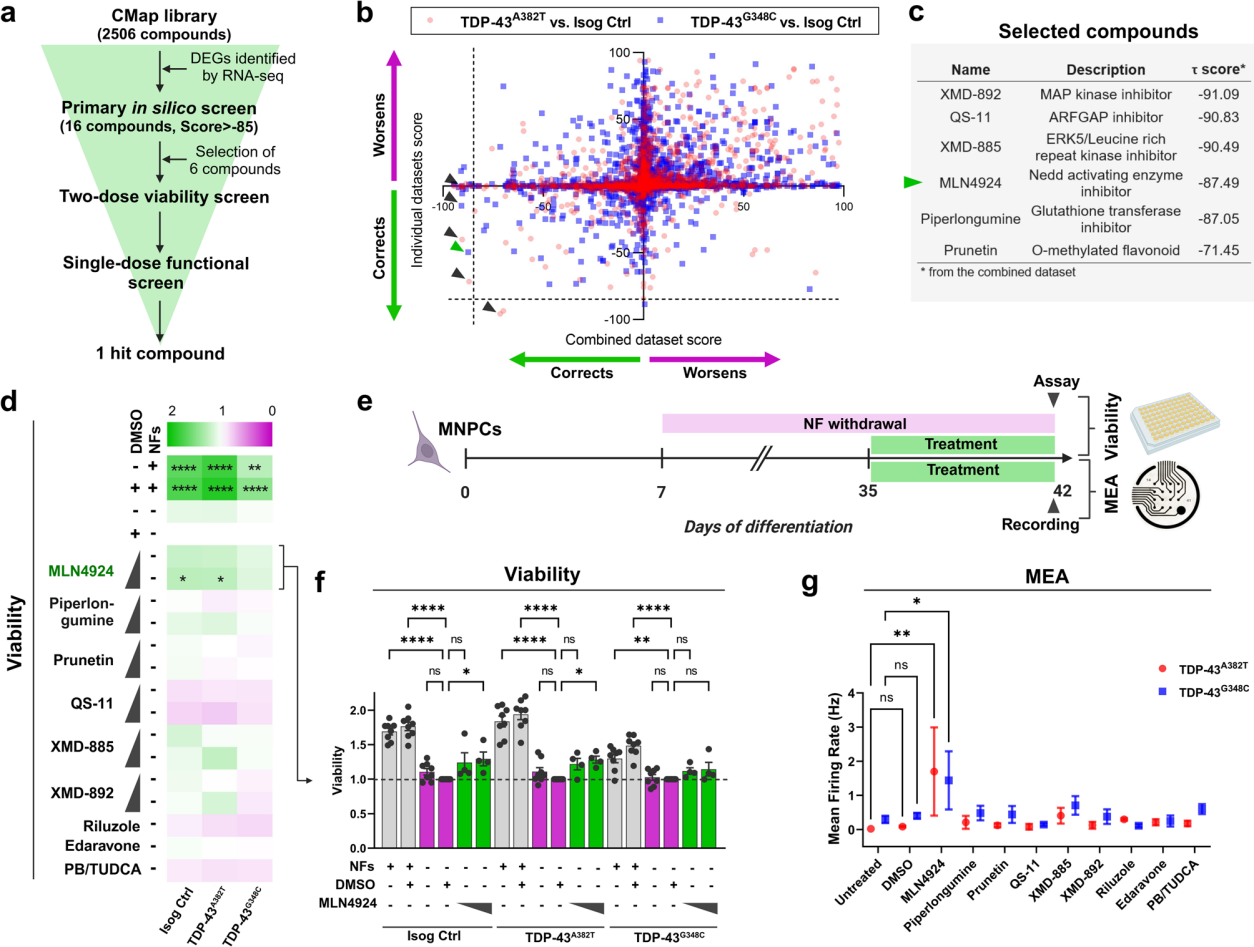
Transcriptome-based *in silico* and *in vitro* phenotypic screens identify one compound that ameliorates MN survival and firing activity. (**a**) Screening funnel used to identify candidate compounds. (**b**) Scatter plot of the τ scores from the individual (y-axis) versus combined (x-axis) datasets gene signatures. Each data point represents one compound of the CMap library. Arrow heads show selected compounds. (**c**) Description and τ scores of selected compounds. (**d**) Heatmap of mean MN survival relative to DMSO control after treatment with compounds (0.1 µM and 1.0 µM) in culture conditions without neurotrophic factors (NFs) supplementation. *n* = 4 independent differentiations. One-way ANOVA with Sidak’s post hoc test. (**e**) Overview of *in vitro* phenotypic screens. (**f**) Bar graph of MN viability after treatment with MLN4924. Data points represent individual wells from *n* = 4 independent differentiations. One-way ANOVA with Sidak’s post hoc test. (**g**) Mean firing rate of MNs treated with candidate compounds (1.0 µM) calculated from per-well mean values from *n* = 4 independent differentiations. Two-way ANOVA with Dunnett’s post hoc test. Data shown as mean ± SEM. *P < 0.05, **P < 0.01, ***P < 0.001, ****P < 0.0001.

Next, we reasoned that mutation-induced gene expression profiles might represent a potential therapeutic opportunity. We posited that transcriptomic alterations may be reflective of underlying disease mechanisms and that normalizing gene expression may have beneficial effects. Thus, we utilized the CMap database^43^ (which comprises of transcriptional profiles induced from thousands of small molecules) to screen for compounds predicted to normalize gene expression changes caused by *TARDBP* mutations toward wild-type levels. We used as inputs our lists of DEGs identified by RNA-seq and obtained as outputs lists of compounds accompanied by their τ scores, indicating the similarity or dissimilarity between the transcriptional changes they induce and the queried DEGs list (Fig. 5a, Supplementary Table S6, Supplementary Data S5). The scores obtained from each queried DEG list were plotted for visualization of hits common between individual and combined datasets (Fig. 5b). Among 16 top-compounds identified (score<-85), we selected 6 candidate compounds from the lower left corner (i.e., predicted to reverse gene expression changes) with high scores in the combined dataset and/or at least one of the individual datasets for further investigation (Fig. 5c). For this prioritization, we took into consideration compound characteristics that could facilitate clinical translation, including currently approved therapeutic uses or investigational uses, known mechanisms of action, and blood-brain barrier penetrance.

### Treatment with hit compound MLN4924 ameliorates MN viability and firing activity

In our next steps, we aimed to determine whether the six selected hit compounds could improve ALS-relevant phenotypic readouts, including MN viability (Fig. 5a). In previous work, we showed that removal of neurotrophic factors (NFs) supplementation from the differentiation medium significantly decreased the viability of MNs irrespective of the cell line or genotype^44^. Thus, we treated MNs cultured without NFs with two concentrations of the candidate compounds (0.1 µM and 1.0 µM) for 6 days to screen for compounds with neuroprotective properties (Fig. 5e). We also included in our compound panel two FDA-approved drugs for the treatment of ALS, namely riluzole and edaravone, as well as the combination of sodium phenylbutyrate (PB) and tauroursodeoxycholic acid (TUDCA), which initially showed promising outcomes in ALS randomized controlled trials^59–61^. Following treatment, one compound (MLN4924) effectively improved viability of isogenic control and TDP-43^A382T^ MNs compared with the vehicle-treated condition at the highest tested concentration, with a similar trend observed in TDP-43^G348C^ MNs (Isog Ctrl: +29.3%, *P* = 0.0352; TDP-43^A382T^: +28.4%, *P* = 0.0472; TDP-43^G348C^: +14.5%, *P* = 0.8681 by one-way ANOVA with Sidak’s multiple comparisons test) (Fig. 5d-f). Perhaps surprisingly, treatments with the ALS drugs did not show similar neuroprotective effects at the concentrations used.

We previously showed using multi-electrode array (MEA) that TDP-43 MNs displayed hypoactivity compared with isogenic control MNs after several weeks in culture^44^. Thus, in addition to viability, we also explored if one or more of the compounds might ameliorate this mutation-induced phenotype. We examined the effects of the six selected hit compounds on neuronal activity in a single-dose screen at the highest concentration (1.0 µM) (Fig. 5a). For this assay, MNs were cultured in complete final differentiation medium with NFs to maintain optimal MN viability for electrophysiological recordings (Fig. 5e). Interestingly, we found that MLN4924 treatment significantly increased the mean firing rate of TDP-43^A382T^ and TDP-43^G348C^ MN cultures compared to untreated cells (TDP-43^A382T^: +1.7 Hz, *P* = 0.0091; TDP-43^G348C^: +1.5 Hz, *P* = 0.0485 by two-way ANOVA with Dunnett’s multiple comparison test) (Fig. 5g). Overall, we demonstrate that combining *in silico* and multi-phenotypic screens approaches enabled the identification of one compound able to improve neuronal viability and activity.

### Components of the NEDDylation pathway are expressed in iPSC-derived MNs and are modulated by MLN4924 treatment

**Fig. 6:**
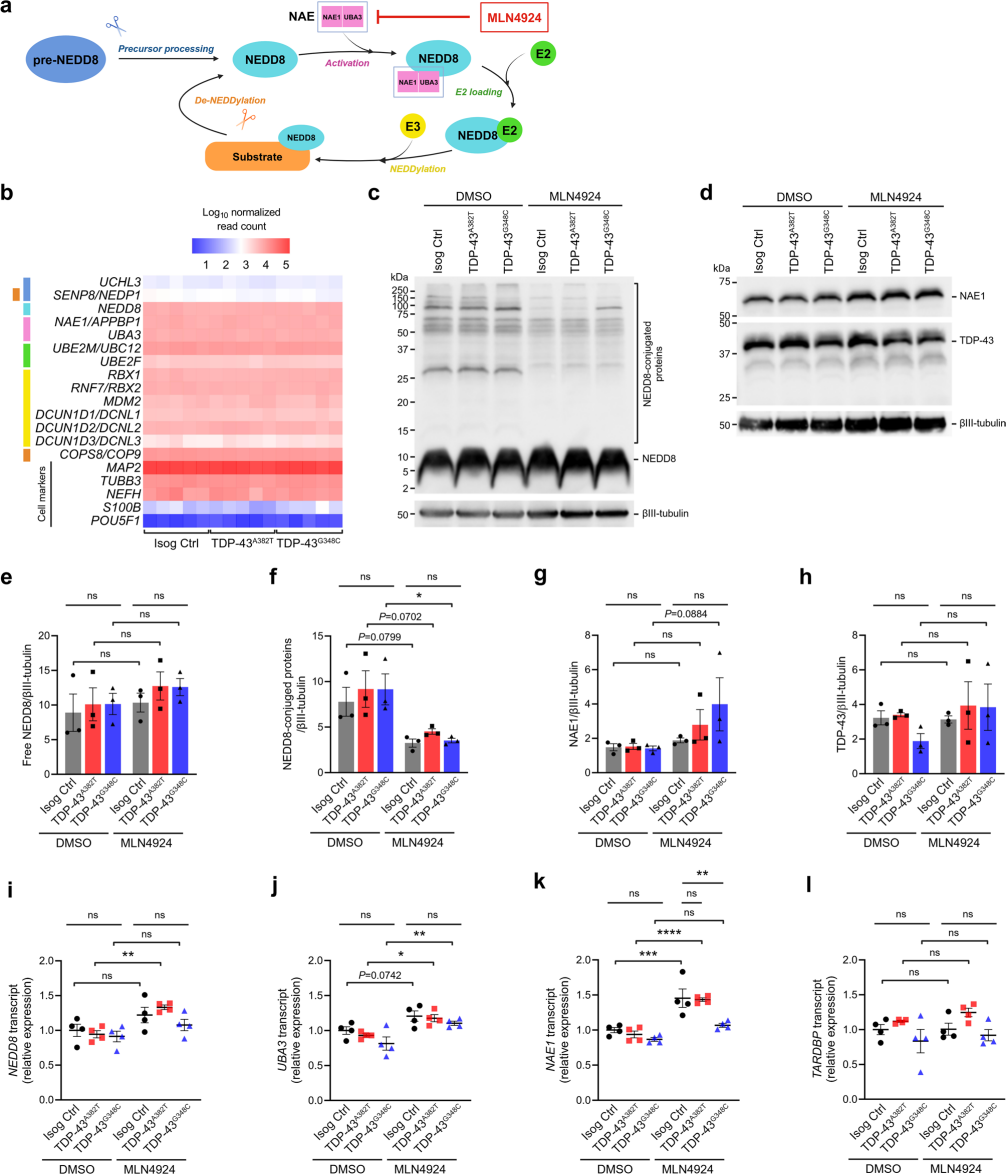
MLN4924 treatment inhibits protein NEDDylation and induces a compensatory upregulation of pathway components. (**a**) Schematic representation of the NEDDylation pathway and target of MLN4924. (**b**) Heatmap of normalized counts of genes involved in the NEDDylation pathway determined by RNA-seq. Color legend refers to NEDDylation steps illustrated in (**a**). Normalized counts of neuronal (*MAP2*, *TUBB3*, *NEFH*), astrocyte (*S100B*) and stem cell (*POU5F1*) markers are displayed for comparison. (**c**–**h**) Representative immunoblots (**c**,**d**) and quantification of free NEDD8 (**e**), NEDD8-conjugated proteins (**f**), NAE1 (**g**), and TDP-43 (**h**) levels in 2-week MNs treated with DMSO or MLN4924 (1.0 µM). βIII-tubulin was used as loading control. *n* = 3 independent experiments. Original uncropped immunoblots are presented in Supplementary Fig. S4. (**i**–**l**) Quantification of relative transcript levels of *NEDD8* (**i**), *UBA3* (**j**), *NAE1* (**k**), and *TARDBP* (**l**) in 4-week MNs treated with DMSO or MLN4924 (1.0 µM) by qPCR. *n* = 4 independent experiments. Data shown as mean ± SEM. Two-way ANOVA with Tukey’s and Sidak’s post hoc tests. *P < 0.05, **P < 0.01, ***P < 0.001, ****P < 0.0001.

To explore the mechanisms underlying MLN4924’s effects in our models, we considered its documented mode of action. MLN4924 is an inhibitor of NEDDylation, a post-translational modification (PTM) similar to ubiquitylation, where an activated NEDD8 is conjugated to a protein substrate^62^. This pathway involves pre-NEDD8 processing enzymes, NEDD8-activating enzyme (NAE) (a heterodimer consisting of NAE1 and UBA3), NEDD8-conjugating enzyme E2, NEDD8-E3 ligases, and de-NEDDylation enzymes (Fig. 6a)^62^. Specifically, MLN4924 inhibits NAE activity, thereby blocking the initial step of this process. Returning to our RNA-seq data, we found that all cell lines expressed numerous genes of the NEDDylation pathway at levels nearly comparable to those of the neuronal markers tubulin β-3 (*TUBB3*) and neurofilament heavy chain (*NEFH*) (Fig. 6b). Western blot experiments revealed similar levels of free NEDD8, NEDD8-conjugated proteins, and NAE1 across cell lines at baseline (DMSO-treated samples) (Fig. 6c-g, Supplementary Fig. S4). We observed a strong trend towards decreased levels of NEDD8-conjugated proteins upon treatment with MLN4924, though only reaching statistical significance in TDP-43^G348C^ MNs (Isog Ctrl *P* = 0.0799; TDP-43^A382T^ *P* = 0.0705; TDP-43^G348C^ *P* = 0.0266 by one-way ANOVA with Sidak’s multiple comparisons test) (Fig. 6c, f, Supplementary Fig. S4). There was also a mild upward trend of free NEDD8 and NAE1 levels post-treatment, mostly in *TARDBP* mutant MNs (Fig. 6c, d,e, g, Supplementary Fig. S4). This effect was more evident at the RNA level, with MLN4924 treatment inducing upregulation of *NEDD8*, *UBE3*, and *NAE1* transcripts to varying degrees across lines (Fig. 6i-k), perhaps reflecting a compensatory response to NEDDylation inhibition. We also analysed the effect of MLN4924 treatment on TDP-43 expression and did not detect any significant changes both at the RNA and protein levels (Fig. 6d, h,l). Overall, our results show that NEDDylation pathway components are expressed in iPSC-derived MNs and appear to be modulated by MLN4924 treatment.

## Discussion

Accumulating evidence implicates RNA dyshomeostasis in the pathobiology of ALS. In particular, the egress of TDP-43 from the nucleus implies a loss of nuclear RNA processing functions at least in advanced stages of ALS, prompting many recent transcriptomic studies in *TARDBP* knockdown cells^16,17,63,64^. However, less attention has so far been given to the impact of ALS-linked *TARDBP* mutations on global RNA metabolism in human iPSC-derived neurons^51,65,66^. Here, we performed whole-transcriptome profiling of MNs differentiated from iPSCs with *TARBDP* mutations coding for TDP-43^A382T^ or TDP-43^G348C^. Our study is the first to provide both mRNA and miRNA expression profiles with integrated mRNA/miRNA analyses, with the aim of increasing our understanding of TDP-43 pathobiology in ALS and identifying molecules able to restore disease-relevant phenotypes.

Our findings highlight significantly overlapping RNA alterations in TDP-43^A382T^ and TDP-43^G348C^ MNs, indicating common pathogenic effects of these mutations on RNA processing. Further comparison of our results with published datasets from other *TARDBP* mutant iPSC-derived MN lines and post-mortem spinal cord specimens revealed shared transcriptomic alterations. Importantly, these results point to several genes previously linked to ALS and other neurodegenerative disorders including frontotemporal dementia (FTD), Parkinson’s disease (PD), and Lewy body dementia (LBD). Among shared DEGs, the long non-coding RNA (lncRNA) *MEG3*, implicated in MN development^67^, was downregulated in both TDP-43^A382T^ and TDP-43^G348C^ datasets and in post-mortem ALS spinal cord specimens. This lncRNA is a known TDP-43 target^19,68^ and loss of TDP-43 binding to *MEG3* was previously observed in brains of patients with FTD^69^. Levels of the PD- and LBD-associated gene *CHCHD2*^70–72^ were also significantly reduced in TDP-43^A382T^ and TDP-43^G348C^ MNs, but were increased in the post-mortem dataset. Coiled-coil-helix-coiled-coil-helix domain containing 2 (encoded by *CHCHD2*) forms a complex with CHCHD10, another nucleus-encoded mitochondrial protein of the CHCHD-containing protein family, known to be genetically linked to ALS and FTD^73–75^. Interestingly, *CHCHD10* was significantly downregulated in TDP-43^A382T^ MNs and post-mortem ALS spinal cords. Previous studies have similarly reported loss of *CHCHD2* expression in TDP-43^M337V^ and TDP-43^Q331K^ iPSC-derived MNs^65^ and decreased *CHCHD10* expression in sporadic ALS patient-derived MNs^76^. Additionally, *CHCHD2* and two other shared DEGs identified here (*PRKCD*, *MYH14*) were predicted to be candidate ALS genes in a machine learning study^77^.

Nonetheless, a considerable number of changes observed in post-mortem samples are not recapitulated in iPSC-derived MNs, as previously observed^49,78^. These differences may reflect the limitations of iPSC-based models such as cell immaturity^79^, the loss of age-related epigenetic marks during reprogramming^80^, and the absence of other cell lineages needed to capture non-cell-autonomous effects. It is also worth noting that TDP-43^A382T^ and TDP-43^G348C^ MNs did not mirror the transcriptional signature of TDP-43 loss-of-function (implicating *STMN2*, *UNC13A*, *ELAVL3*, *PFKP*, *RCAN1*, *SELPLG*) established by previous knockdown studies and other ALS iPSC models^16,17,51,63,64,81,82^, perhaps consistent with the preserved nuclear localization of TDP-43 in our cells^44^. Thus, it appears that the TDP-43^A382T^ and TDP-43^G348C^ variants impact RNA processing by mechanisms distinct from nuclear clearance, as noted in previous *TARDBP*/*Tardbp* mutant mouse models^36–39^. Like these studies, we posit that the observed mutation-induced transcriptome profiles may reflect early RNA perturbations in the pathogenesis of TDP-43-ALS, preceding nuclear TDP-43 loss.

We explored the possibility that the *TARDBP* mutations studied could impact gene expression through altered miRNA biogenesis. miRNAs are highly expressed in the CNS and have been shown to be dysregulated in ALS patient specimens^83–89^. We found a marked overlap in dysregulated miRNAs in both mutant MNs with concordant fold changes and directionality, some of which were previously linked to ALS. Specifically, dysregulation of miR-370-3p, miR-409-3p, and miR-495-3p was documented in iPSC-derived MNs with homozygous FUS^P517L^ mutations^90^. Other dysregulated miRNAs, namely miR-432-5p, miR-323a-3p and miR-453, were predicted here to target *ALS2*, associated with juvenile ALS^91,92^. Additionally, the ALS-associated gene *SARM1*^93,94^ was predicted to be regulated by miR-432-5p, mir-370-3p, miR-453, miR-495-3p, and miR-654-5p, all miRNAs that were downregulated in our study. In agreement with the studies cited above, our work may support the view that miRNAs contribute to ALS pathophysiology.

Strikingly, most altered miRNAs mapped to the 14q32 miRNA cluster, implying that their expression is regulated by a common transcriptional or epigenetic process. Members of miRNAs clusters typically share similar biological functions by targeting the same sets of genes or functionally related genes of the same pathways^95^. The miRNAs of the 14q32 cluster (also known as the miR379–410 or C14MC cluster) are highly conserved, brain-enriched miRNAs which are involved in many aspects of neuronal development and function including neuronal migration, neurite formation, and synaptic plasticity^96,97^. Accordingly, we found several differentially regulated miRNA/mRNA pairs implicating protocadherins, which play key roles in neural circuit formation^55^. The miRNAs of this cluster were also previously associated with brain disorders including epilepsy^98,99^, schizophrenia^100,101^, autism spectrum disorder^102,103^, and brain cancers^104,105^. Interestingly, miRNAs of this cluster were shown to be selectively exported via exosomes^106^, and several have been detected in the serum^107^, suggesting they could constitute promising biomarkers. In fact, levels of the 14q32-encoded miRNA miR-127-3p were altered in MNs in this study as well as in the serum of sporadic and familial ALS patients in two previous studies^108,109^.

In addition to biomarkers, miRNAs are also increasingly regarded as potential therapeutic targets, given that miRNAs can modulate hundreds of targets simultaneously^110^. In future work, individual miRNA mimics or synthetic miRNA clusters^111^ may be employed to modulate levels of dysregulated miRNAs and their targets to assess phenotypic rescue. Such therapeutic strategies capable of acting more broadly on multiple cellular pathways, rather than on a few targets, may be more likely to have a clinically significant impact on the disease course. However, an important consideration for miRNA replacement therapy is the potential for undesirable off-target effects, which can be hard to predict or avoid^112^ as well as the invasive nature of the treatments (i.e., intrathecal injections) that are not without risk for patients.

Small orally bioavailable molecules able to improve the function and viability of MNs may be promising ALS therapies, given their ease of administration. Thus, we leveraged the transcriptomic profiles of TDP-43 MNs to search for compounds predicted to correct gene expression using the CMap database. This drug discovery strategy was previously applied to several human conditions including aortic valve disease^113,114^, non-alcoholic steatohepatitis^115^, epilepsy^116–118^, schizophrenia^119^, neurodevelopmental disorders^42^, and FTD^120^, yet remains largely unexplored in the ALS field. Among top-scoring compounds, several had pharmacological effects that could potentially be relevant for the treatment of ALS. Some were known to act in the CNS for the treatment of neurological symptoms (e.g., neuroleptic/antipsychotic, anticonvulsant) and/or were ligands of neurotransmitter receptors (e.g., dopamine receptor, serotonin receptor), namely sulpiride, QS-11, GR-46,611, and CS-110266. Interestingly, sulpiride is a neuroleptic and in a previous large-scale chemical screen conducted in TDP-43^A315T^ worms and TDP-43^G348C^ zebrafish, all the compounds identified as hits were also neuroleptics^121^. Additionally, while there is evidence of increased MAP kinase signaling in ALS patients as well as in animal and iPSC models^49,66,122–124^, two compounds identified here were MAP kinase inhibitors (XMD-892, XMD-885). Finally, XMD-892^125^ and other compounds (tacedinaline^126–128^, piperlongumine^129,130^, prunetin^131^) have also been investigated for their antioxidant, anti-inflammatory, anti-ischemic, neuroprotective, and/or antitumour properties. *In silico* compound prediction was followed by two *in vitro* screens based on viability and hypoactivity phenotypes described in previous work^44^. We tested a panel of 6 top-scoring compounds along with three ALS drugs: riluzole, edaravone, and PB/TUDCA. These efforts led to the identification of the NEDDylation inhibitor MLN4924, which effectively improved neuronal viability and firing activity. Finally, we showed that components of the NEDDylation pathway are enriched in MNs and that MLN4924 treatment reduces levels of NEDD8-conjugated proteins, thereby demonstrating target engagement.

Protein NEDDylation has been positively implicated in neuronal development, synapse formation, and neurotransmission^132–136^. However, overactivation of NEDDylation has been linked to neuronal apoptosis^137^ and brain malignancies^138,139^. NEDD8 has been detected within ubiquitin-positive inclusions in several neurodegenerative diseases including ALS, ALS/FTD, PD, and Alzheimer’s disease^140^. Furthermore, a single nucleotide polymorphism (SNP) in *DCUN1D1*, a component of the NEDDylation pathway^141^, was found to increase the risk of developing FTD by about 4-fold^142^. MLN4924 is a first-in-class drug to have entered phase II and III clinical trials for the treatment of various cancers^143^. As it is brain-penetrant and generally well tolerated by patients^143^, its therapeutic effects are also being considered for the treatment of neurological disorders. MLN4924 was shown to be neuroprotective against ischemic injury in a mouse model of ischemic stroke^144^. This compound was also reported to attenuate reactive oxygen species production and cell damage and death induced by H_2_O_2_ stress in rat primary neurons^145^. Recently, genetic modulation of the NEDDylation pathway ameliorated motor phenotypes in nematode models of ALS expressing mutant forms of *SOD1* and *C9ORF72*^146^. Here, we showed that MLN4924 exerted desirable effects in *TARDBP* mutant iPSC-derived MNs, although its mechanisms of action in our models remains to be defined. Given that PTMs can regulate protein-protein interactions and liquid-liquid phase separation behavior^147^, we speculate that NEDDylation inhibition by MLN4924 may modulate the formation of RNP condensates, a process central to several RNA processing functions of TDP-43. In fact, modulating the NEDDylation pathway has been linked to the assembly and disassembly of stress granules^146,148,149^. NEDDylation has also been shown to promote nuclear protein aggregation under proteotoxic stress conditions^150^. These findings and the apparent neuroprotective effects of MLN4924 in our iPSC models highlight the therapeutic potential of this compound and calls for further investigation into the roles of protein NEDDylation in TDP-43 biology and ALS.

In summary, this study highlights the value of whole-transcriptome profiling for shedding light on disease-relevant perturbed pathways, and for identifying potential therapeutic candidates through a transcriptome reversal paradigm. *In silico* prediction of MLN4924 translated into improvement of disease-relevant phenotypes *in vitro* in our models, demonstrating the potential of this strategy for drug discovery in ALS and other neurodegenerative diseases.

## Methods

All methods were performed in accordance with the relevant guidelines and regulations.

### iPSC lines and culture

The use of human cells in this study was approved by McGill University Health Center Research Ethics Board (DURCAN_iPSC / 2019–5374). The *TARDBP* knock-in cell lines used in this study were generated from the control cell line AIW002-02, as detailed in our earlier work^44^. This control cell line was reprogrammed from peripheral blood mononuclear cells (PBMCs) of a 37-year-old Caucasian male, as previously described^151^. Informed consent was obtained from this subject. A summary of the iPSC lines used can be found in Supplementary Table S1. iPSCs were maintained on dishes coated with Matrigel (Corning Millipore; Cat#354277) in mTeSR1 (StemCell Technologies; Cat#85850) with daily media change and passaged at 80% confluence using Gentle Cell Dissociation Reagent (StemCell Technologies; Cat#07174). Cultures were routinely tested for mycoplasma using the MycoAlert Mycoplasma Detection kit (Lonza; Cat#LT07-318).

### Differentiation of MNs from iPSCs

IPSCs were first induced into MN progenitor cells (MNPCs) by dual-SMAD signaling inhibition and ventral neural patterning as previously described^152^. Banked MNPCs were thawed onto dishes coated with 10 µg/ml Poly-L-ornithine (PLO) (Sigma-Aldrich; Cat#P3655) and 5 µg/ml laminin (Sigma-Aldrich; Cat#L2020) in “expansion medium” composed of basic neural medium (1:1 mixture of DMEM/F12 medium (Gibco; Cat#10565–018) and Neurobasal medium (Life Technologies; Cat#21103–049), 0.5X N2 (Life Technologies; Cat#17502–048), 0.5X B27 (Life Technologies; Cat#17504–044), 0.5X GlutaMAX (Gibco, Cat#35050-061), 1X antibiotic-antimycotic (Gibco, Cat#15240–062), and 100 µM ascorbic acid (Sigma-Aldrich; Cat#A5960)) supplemented with 3 µM CHIR99021 (Selleckchem; Cat#S2924), 2 µM SB431542 (Selleckchem; Cat#S1067), 2 µM DMH1 (Selleckchem; Cat#S7146), 0.1 µM retinoic acid (Sigma-Aldrich; Cat#R2625), 0.5 µM purmorphamine (Sigma-Aldrich; Cat#SML-0868), 0.5 mM valproic acid (Sigma-Aldrich; Cat#P4543)) and 10 µM ROCK inhibitor Y-27632 (Selleckchem; Cat#S1049) (for the first 24 h), and were allowed to recover up to 6 days with medium fully changed every other day.

For final MN differentiation, MNPCs were dissociated as single cells with Accutase (StemCell Technologies; Cat#07922) and were seeded on dishes coated with 10 µg/ml PLO and 5 µg/ml laminin (Life Technologies; Cat#23017-015) in “final differentiation medium” composed of basic neural medium supplemented with 0.5 µM retinoic acid, 0.1 µM purmorphamine, 0.1 µM Compound E (StemCell Technologies; Cat#73954), and 10 ng/ml insulin-like growth factor-1 (IGF-1, Peprotech; Cat#100–11), brain-derived neurotrophic factor (BDNF, Peprotech; Cat#450–02) and ciliary neurotrophic factor (CNTF, Peprotech; Cat#450–13)), with half-changes at least every other week. Alternatively, for viability and MEA experiments, MNPCs were first passaged at a 1:3 ratio in “priming medium” composed of basic neural medium supplemented with 0.5 µM retinoic acid and 0.1 µM purmorphamine with medium changed every other day for 6 days before they were plated for final differentiation as described above.

### Library preparation and sequencing

Total RNA was extracted from MN cultures using the miRNeasy micro kit (Qiagen; Cat#217004) with DNase treatment (Qiagen; Cat#79256) following the manufacturer’s instructions. Samples with RNA integrity numbers above 6.5 determined by a Bioanalyzer (Agilent) were used for library preparation using the NEB mRNA stranded library preparation kit (for RNA-seq) and the NEB small RNA library preparation kit (for small RNA-seq). Libraries were prepared and sequenced at Genome Québec (Montreal, Canada) on a Illumina NovaSeq 6000 sequencing system with paired-end 100 bp (PE100) strategy.

### RNA-seq analysis

Sequencing files were analyzed using the GenePipes RNA-seq pipeline 3.6.0^153^. Briefly, raw reads were clipped for adapter sequence, trimmed for minimum quality (Q30) in 3’ and filtered for minimum length of 32 bp using Trimmomatic^154^. Surviving read pairs were aligned to the human genome assembly hg38 by the ultrafast universal RNA-seq aligner STAR^155^ using the recommended two passes approach. Aligned RNA-seq reads were assembled into transcripts and their relative abundance was estimated using Cufflinks^156^. Differential expression analyses were conducted using DESeq2^157^ with a false discovery rate of 5% with no cut-off on log fold-change. Volcano plots were created using the Molecular and Genomics Informatics Core (MaGIC) Volcano Plot Tool, available at https://volcano.bioinformagic.tools/.

### miRNA profiling

Sequencing datasets were analyzed with a modified version of the workflow proposed by Potla et al.^158^. We adapted this workflow to analyze reads without UMI’s, single end and paired end readsets, and multiple readsets per sample (this workflow is available at https://github.com/neurobioinfo/miRNA_workflow). After quality control with FastQC (http://www.bioinformatics.babraham.ac.uk/projects/fastqc/*)*, we used cutadapt to remove reads shorter than 15 nt and larger than 32 nt. We used Bowtie v1.3^159^ to align readsets to the miRBase database v22^160–164^ and to the human reference genome v104. In order to maximize the number of alignments and to detect rare miRNAs, we used a seed length 20 (Bowtie option -l 20) and allowed 3 mismatches within the seed (Bowtie option -n 3). MiRNAs aligned to miRBase were counted with Samtools idxstats^165^. MiRNAs aligned to the reference genome were filtered with TAGBAM from BEDTools^166^ and counted with a locally developed script that optimizes counting time (https://github.com/neurobioinfo/miRNA_workflow*).* Finally, the local workflow adds miRNAs aligned to miRBase to those aligned to the reference genome for each sample, and generates a miRNA counts matrix with all samples counts. Before differential expression analyses with the R package DESeq2^157^, the count matrix was pre-filtered to keep miRNAs having at least 10 counts. DESeq2 estimates dispersions using the Cox-Reid method^167^. It uses negative binomial GLM fitting for log2 fold changes estimation and the Wald statistics for hypothesis testing. We used a false discovery rate of 5% with no cut-off on log2 fold change.

### Gene ontology and KEGG enrichment analysis

Terms from the Gene Ontology were tested for enrichment with the ShinyGO online tool, available at http://bioinformatics.sdstate.edu/go/.

### miRNA target prediction

Prediction of miRNA targets was performed using miRGate^54^, which uses five different public prediction algorithms. We considered target genes predicted by at least two algorithms.

### Quantitative PCR

Expression levels of genes of interest were quantified using quantitative PCR (qPCR). cDNA synthesis was performed with 500 ng of total RNA using the M-MLV Reverse Transcriptase kit (Thermo Fischer Scientific; Cat#28025013) in a total volume of 40 µL. To quantify expression levels of selected miRNAs, cDNA synthesis was performed using 10 ng of total RNA with the TaqMan^®^ Advanced miRNA cDNA Synthesis Kit following the manufacturer’s instructions. Real-time qPCR reactions were set up in triplicates with the Applied Biosystems Applied Biosystems SYBR green (Applied Biosystems; Cat#A25778) or TaqMan^®^ Fast Advanced Master Mix (Applied Biosystems; Cat#A44360) and run on a QuantStudio 5 Real-Time PCR system (Applied Biosystems Cat#A28140). Primers and TaqMan^®^ probes references are provided in Supplementary Table S2. Levels of the endogenous control miRNA hsa-miR-191-5p^168^ and the geometric mean of the endogenous control genes *18 S* (*RN18S1*), *HPRT1*, *PPIA* and *POLR2A* were used for normalization. Normalized expression was displayed relative to the relevant control condition.

### *In silico* screen with the CMap database

The lists of down/upregulated genes of each contrast were used as inputs in the CMap database^43^ to assess the similarity (or dissimilarity) between the query gene set and gene expression profiles induced by a library of small molecules. The obtained output is a list of the small molecules rank-ordered by their CMap connectivity score (τ), a normalized metric ranging from − 100 to 100. A negative score indicates opposing profiles between the small molecule and the query gene set, thereby predicting the small molecule to reverse gene expression changes. In contrast, a positive score indicates similar profiles.

### Two-dose viability screen

MNPCs were plated as 15,000 cells per well in opaque white optical 96-well plates coated with PLO/laminin. Cells were cultured in final differentiation medium for one week, after which they were treated with 1 µM cytosine arabinoside (AraC, Sigma-Aldrich; Cat#C6645) overnight (~ 17 h) to eliminate any residual proliferating cells. The next day, medium was fully changed to final differentiation medium with or without supplementation of neurotrophic factors (NFs) (i.e., BDNF, CNTF, and IGF-1), with half-changes every other week until initiation of the assay. After 5 weeks of final differentiation, MNs were treated with the candidate compounds MLN4924, piperlongumine, prunetin, QS-11, XMD-885, XMD-892 at the final concentrations of 0.1 µM and 1.0 µM, or with 3 µM riluzole, 0.025 µM edaravone or the combination of 250 µM sodium phenylbutyrate (PB)/10 µM tauroursodeoxycholic acid (TUDCA) for 6 days with medium renewed every second day. After treatment, viability was assessed with an ATP-based chemiluminescent assay (Cell Titer-Glo^®^, Promega; Cat#G7570) following the manufacturer’s instructions. The luminescence readings were acquired using a GloMax Microplate Reader (Promega). Raw luminescence values were normalized to the vehicle condition (mean of DMSO-treated wells, medium without NFs) for each cell line. The percentage increase in viability post-treatment reported in text represents the difference in normalized luminescence values between DMSO- and MLN4924-treated cells.

### Single-dose functional screen

To assess the effect of compounds on neuronal activity, treatments were performed in MNs differentiated for 5 weeks in 24-well MEA plates (Axion Biosystems) in complete final differentiation medium. MNs were treated with candidate compounds for 6 days at a final concentration of 1.0 µM or with the ALS drugs as described above. After treatment, MEA recordings and analyses were carried out as previously described^44^. Briefly, MNs were incubated in freshly prepared pre-warmed carbonated artificial cerebrospinal fluid (aCSF)^169^ for at least 1 h. Spontaneous activity was recorded for 5 min in a Maestro Edge MEA system (Axion Biosystems) and the AxiS v.66,465 software (Axion Biosystems).

### Western blot

Cell pellets were collected from MN cultures treated with DMSO or 1.0 µM MLN4924 for 6 days as described above at 2 weeks post-plating of progenitors. Proteins were extracted using ice-cold RIPA buffer (Millipore; Cat#20–188) supplemented with protease inhibitors (Roche; Cat#11697498001) and phosphatase inhibitors (Roche; Cat#04906837001), as previously described^44^. Protein concentrations were determined using the DC Protein Assay (Bio-Rad; Cat#5000111). A total of 20 µg of protein was resolved by 10% or 13% SDS/PAGE and transferred to nitrocellulose membranes using a Trans-Blot Turbo Transfer System (Bio-Rad). After transfer, membranes were blocked in 5% BSA in TBS-T 0.1% for 1 h at room temperature and incubated with primary antibodies in blocking solution overnight at 4 °C. After three washes with TBS-T 0.1%, membranes were incubated with horseradish peroxidase (HRP)-conjugated secondary antibodies (1:10000) in blocking solution for 1 h at room temperature. Blots were developed with Clarity Western ECL Substrate (Bio-Rad; Cat#170–5061) and pictures were acquired with a ChemiDoc MP Imaging System (Bio-Rad). Semiquantitative analysis of western blots was performed with the Image Lab 6.0.1 software (Bio-Rad), using βIII-tubulin as a loading control. The following primary antibodies were used: mouse anti-βIII-tubulin (Millipore; Cat#MAB5564; 1:40000), rabbit anti-TDP-43 (C-terminal) (Proteintech; Cat#12892-1-AP; 1:4000), rabbit anti-NEDD8 (Cell Signaling Technology, Cat#2754; 1:1000), and rabbit anti-NAE1 (Cell Signaling Technology, Cat#14321; 1:2000). The following secondary antibodies were used: HRP-conjugated anti-Mouse (Jackson Immunoresearch, Cat#115-035-003) and HRP-conjugated anti-Rabbit (Jackson Immunoresearch, Cat#111-035-144).

### Statistics

Biological replicates were defined as independent differentiations. Hypergeometric testing was performed to compare the lists of genes and miRNAs using the following web app: http://nemates.org/MA/progs/overlap_stats.html. For qPCR experiments, Grubbs’ test was used to determine significant outliers. Statistical analyses were performed with the GraphPad Prism software. Data distribution was assumed to be normal although this was not formally tested. Differences between mutants and control were analyzed using one-way or two-way analysis of variance (ANOVA) tests. Means and standard errors of the mean were used for data presentation. Significance was defined as *P* < 0.05.

## Electronic supplementary material

Below is the link to the electronic supplementary material.

Supplementary Material 1

Supplementary Material 2

Supplementary Material 3

Supplementary Material 4

Supplementary Material 5

Supplementary Material 6

## Data Availability

The data supporting the findings of this study are available from the corresponding authors on reasonable request. The RNA-seq and small RNA-seq datasets generated during the current study are available in the Gene Expression Omnibus (GEO) repository (http://www.ncbi.nlm.nih.gov/geo) under the accession numbers GSE277084 and GSE277085.

## References

[1] Lechtzin, N., Wiener, C. M., Clawson, L., Chaudhry, V. & Diette, G. B. Hospitalization in amyotrophic lateral sclerosis. *Neurology* **56**, 753–757 (2001). 11274310 10.1212/wnl.56.6.753

[2] Moura, M. C. et al. Prognostic factors in amyotrophic lateral sclerosis: A population-based study. *PLoS One*. **10**, e0141500 (2015). 26517122 10.1371/journal.pone.0141500 PMC4627754

[3] Bensimon, G., Lacomblez, L. & Meininger, V. A controlled trial of riluzole in amyotrophic lateral sclerosis. *N Engl. J. Med.* **330**, 585–591 (1994). 8302340 10.1056/NEJM199403033300901

[4] Lacomblez, L., Bensimon, G., Meininger, V., Leigh, P. & Guillet, P. Dose-ranging study of riluzole in amyotrophic lateral sclerosis. *Lancet* **347**, 1425–1431 (1996). 8676624 10.1016/s0140-6736(96)91680-3

[5] Miller, R. G., Mitchell, J. D. & Moore, D. H. Riluzole for amyotrophic lateral sclerosis (ALS)/motor neuron disease (MND). *Cochrane Database Syst. Rev.*10.1002/14651858.CD001447.pub3 (2012). 11687111 10.1002/14651858.CD001447

[6] Abe, K. et al. Safety and efficacy of Edaravone in well defined patients with amyotrophic lateral sclerosis: a randomised, double-blind, placebo-controlled trial. *Lancet Neurol.* **16**, 505–512 (2017). 28522181 10.1016/S1474-4422(17)30115-1

[7] Sakata, T., Palumbo, J., Akimoto, M. & Tanaka, M. A long-term safety and efficacy extension study of patients diagnosed with amyotrophic lateral sclerosis (ALS) and treated with Edaravone (MCI-186) (P3.192). *Neurology* **86**, 505–512 (2016).26747886

[8] Miller, T. et al. Phase 1–2 trial of antisense pligonucleotide Tofersen for SOD1 ALS. *N Engl. J. Med.* **383**, 109–119 (2020). 32640130 10.1056/NEJMoa2003715

[9] Miller, T. M. et al. Trial of antisense oligonucleotide Tofersen for SOD1 ALS. *N Engl. J. Med.* **387**, 1099–1110 (2022). 36129998 10.1056/NEJMoa2204705

[10] Meyer, T. et al. Neurofilament light-chain response during therapy with antisense oligonucleotide Tofersen in SOD1-related ALS: treatment experience in clinical practice. *Muscle Nerve*. **67**, 515–521 (2023). 36928619 10.1002/mus.27818

[11] Arai, T. et al. TDP-43 is a component of ubiquitin-positive tau-negative inclusions in frontotemporal Lobar degeneration and amyotrophic lateral sclerosis. *Biochem. Biophys. Res. Commun.* **351**, 602–611 (2006). 17084815 10.1016/j.bbrc.2006.10.093

[12] Mackenzie, I. R. A. et al. Pathological TDP-43 distinguishes sporadic amyotrophic lateral sclerosis from amyotrophic lateral sclerosis with SOD1 mutations. *Ann. Neurol.* **61**, 427–434 (2007). 17469116 10.1002/ana.21147

[13] Neumann, M. et al. Ubiquitinated TDP-43 in frontotemporal Lobar degeneration and amyotrophic lateral sclerosis. *Sci. (80-)*. **314**, 130–133 (2006). 10.1126/science.1134108 17023659

[14] Lill, C. M., Abel, O., Bertram, L. & Al-Chalabi, A. Keeping up with genetic discoveries in amyotrophic lateral sclerosis: the ALSoD and ALSGene databases. *Amyotroph. Lateral Scler.* **12**, 238–249 (2011). 21702733 10.3109/17482968.2011.584629

[15] Abel, O., Powell, J. F., Andersen, P. M. & Al-Chalabi, A. ALSoD: A user-friendly online bioinformatics tool for amyotrophic lateral sclerosis genetics. *Hum. Mutat.*10.1002/humu.22157 (2012). 22753137 10.1002/humu.22157

[16] Brown, A. et al. TDP-43 loss and ALS-risk SNPs drive mis-splicing and depletion of UNC13A. *Nature* **603**, 131–137 (2022). 35197628 10.1038/s41586-022-04436-3 PMC8891020

[17] Ma, X. R. et al. TDP-43 represses cryptic exon inclusion in the FTD–ALS gene UNC13A. *Nature* **603**, 124–130 (2022). 35197626 10.1038/s41586-022-04424-7 PMC8891019

[18] Prudencio, M. et al. Distinct brain transcriptome profiles in C9orf72-associated and sporadic ALS. *Nat. Neurosci.* **18**, 1175–1182 (2015). 26192745 10.1038/nn.4065 PMC4830686

[19] Krach, F. et al. Transcriptome–pathology correlation identifies interplay between TDP-43 and the expression of its kinase CK1E in sporadic ALS. *Acta Neuropathol.* **136**, 405–423 (2018). 29881994 10.1007/s00401-018-1870-7 PMC6215775

[20] Jiang, Y. M. et al. Gene expression profile of spinal motor neurons in sporadic amyotrophic lateral sclerosis. *Ann. Neurol.* **57**, 236–251 (2005). 15668976 10.1002/ana.20379

[21] Rabin, S. J. et al. Sporadic ALS has compartment-specific aberrant exon splicing and altered cell-matrix adhesion biology. *Hum. Mol. Genet.* **19**, 313–328 (2009). 19864493 10.1093/hmg/ddp498 PMC2796893

[22] Humphrey, J. et al. Integrative transcriptomic analysis of the amyotrophic lateral sclerosis spinal cord implicates glial activation and suggests new risk genes. *Nat. Neurosci.* **26**, 150–162 (2023). 36482247 10.1038/s41593-022-01205-3

[23] Dols-Icardo, O. et al. Motor cortex transcriptome reveals microglial key events in amyotrophic lateral sclerosis. *Neurol. Neuroimmunol. Neuroinflamm.* **7**, (2020). 10.1212/NXI.0000000000000829 PMC7371375 32669313

[24] Ou, S. H., Wu, F., Harrich, D., García-Martínez, L. F. & Gaynor, R. B. Cloning and characterization of a novel cellular protein, TDP-43, that binds to human immunodeficiency virus type 1 TAR DNA sequence motifs. *J. Virol.* **69**, 3584–3596 (1995). 7745706 10.1128/jvi.69.6.3584-3596.1995 PMC189073

[25] Buratti, E. Nuclear factor TDP-43 and SR proteins promote in vitro and in vivo CFTR exon 9 skipping. *EMBO J.* **20**, 1774–1784 (2001). 11285240 10.1093/emboj/20.7.1774 PMC145463

[26] Buratti, E. et al. Nuclear factor TDP-43 can affect selected microRNA levels. *FEBS J.* **277**, 2268–2281 (2010). 20423455 10.1111/j.1742-4658.2010.07643.x

[27] Kawahara, Y. & Mieda-Sato, A. TDP-43 promotes microRNA biogenesis as a component of the drosha and Dicer complexes. *Proc. Natl. Acad. Sci.* **109**, 3347–3352 (2012). 22323604 10.1073/pnas.1112427109 PMC3295278

[28] Fallini, C., Bassell, G. J. & Rossoll, W. The ALS disease protein TDP-43 is actively transported in motor neuron axons and regulates axon outgrowth. *Hum. Mol. Genet.* **21**, 3703–3718 (2012). 22641816 10.1093/hmg/dds205 PMC3406762

[29] Alami, N. H. et al. Axonal transport of TDP-43 mRNA granules is impaired by ALS-causing mutations. *Neuron* **81**, 536–543 (2014). 24507191 10.1016/j.neuron.2013.12.018 PMC3939050

[30] Wang, I. F., Wu, L. S., Chang, H. Y. & Shen, C. K. J. TDP-43, the signature protein of FTLD-U, is a neuronal activity-responsive factor. *J. Neurochem*. **105**, 797–806 (2008). 18088371 10.1111/j.1471-4159.2007.05190.x

[31] Casafont, I., Bengoechea, R., Tapia, O., Berciano, M. T. & Lafarga, M. TDP-43 localizes in mRNA transcription and processing sites in mammalian neurons. *J. Struct. Biol.* **167**, 235–241 (2009). 19539030 10.1016/j.jsb.2009.06.006

[32] Pérez-Berlanga, M. et al. Loss of TDP‐43 oligomerization or RNA binding elicits distinct aggregation patterns. *EMBO J.* **42**, e111719 (2023). 37431963 10.15252/embj.2022111719 PMC10476175

[33] Colombrita, C. et al. TDP-43 is recruited to stress granules in conditions of oxidative insult. *J. Neurochem*. **111**, 1051–1061 (2009). 19765185 10.1111/j.1471-4159.2009.06383.x

[34] Liu-Yesucevitz, L. et al. Tar DNA binding protein-43 (TDP-43) associates with stress granules: analysis of cultured cells and pathological brain tissue. *PLoS One*. **5**, e13250 (2010). 20948999 10.1371/journal.pone.0013250 PMC2952586

[35] McDonald, K. K. et al. TAR DNA-binding protein 43 (TDP-43) regulates stress granule dynamics via differential regulation of G3BP and TIA-1. *Hum. Mol. Genet.* **20**, 1400–1410 (2011). 21257637 10.1093/hmg/ddr021

[36] Watanabe, S. et al. ALS-linked TDP-43M337V knock-in mice exhibit splicing deregulation without neurodegeneration. *Mol. Brain*. **13**, 8 (2020). 31959210 10.1186/s13041-020-0550-4 PMC6971932

[37] White, M. A. et al. TDP-43 gains function due to perturbed autoregulation in a Tardbp knock-in mouse model of ALS-FTD. *Nat. Neurosci.* **21**, 552–563 (2018). 29556029 10.1038/s41593-018-0113-5 PMC5884423

[38] Arnold, E. S. et al. ALS-linked TDP-43 mutations produce aberrant RNA splicing and adult-onset motor neuron disease without aggregation or loss of nuclear TDP-43. *Proc. Natl. Acad. Sci.* **110**, e736–e745 (2013). 10.1073/pnas.1222809110 PMC3581922 23382207

[39] Fratta, P. et al. Mice with endogenous TDP-43 mutations exhibit gain of splicing function and characteristics of amyotrophic lateral sclerosis. *EMBO J.* **37**, 1–15 (2018). 29764981 10.15252/embj.201798684 PMC5983119

[40] Marques, R. F. et al. Motor neuron translatome reveals deregulation of SYNGR4 and PLEKHB1 in mutant TDP-43 amyotrophic lateral sclerosis models. *Hum. Mol. Genet.* **29**, 2647–2661 (2020). 32686835 10.1093/hmg/ddaa140 PMC7530531

[41] Gordon, D. et al. Single-copy expression of an amyotrophic lateral sclerosis-linked TDP-43 mutation (M337V) in BAC Transgenic mice leads to altered stress granule dynamics and progressive motor dysfunction. *Neurobiol. Dis.* **121**, 148–162 (2019). 30290270 10.1016/j.nbd.2018.09.024

[42] Dhindsa, R. S., Zoghbi, A. W., Krizay, D. K., Vasavda, C. & Goldstein, D. B. A transcriptome-based drug discovery paradigm for neurodevelopmental disorders. *Ann. Neurol.* **89**, 199–211 (2021). 33159466 10.1002/ana.25950 PMC8122510

[43] Lamb, J. et al. The connectivity map: using gene-expression signatures to connect small molecules, genes, and disease. *Sci. (80-)*. **313**, 1929–1935 (2006). 10.1126/science.1132939 17008526

[44] Lépine, S. et al. Homozygous ALS-linked mutations in TARDBP/TDP-43 lead to hypoactivity and synaptic abnormalities in human iPSC-derived motor neurons. *iScience* **27**, 109166 (2024). 38433895 10.1016/j.isci.2024.109166 PMC10905001

[45] Thaler, J. P. et al. A postmitotic role for Isl-Class LIM homeodomain proteins in the assignment of visceral spinal motor neuron identity. *Neuron* **41**, 337–350 (2004). 14766174 10.1016/s0896-6273(04)00011-x

[46] Thiry, L., Hamel, R., Pluchino, S., Durcan, T. & Stifani, S. Characterization of human iPSC-derived spinal motor neurons by single-cell RNA sequencing. *Neuroscience*10.1016/j.neuroscience.2020.04.041 (2020). 32380268 10.1016/j.neuroscience.2020.04.041

[47] De Santis, R. et al. FUS mutant human motoneurons display altered transcriptome and microRNA pathways with implications for ALS pathogenesis. *Stem Cell. Rep.* **9**, 1450–1462 (2017). 10.1016/j.stemcr.2017.09.004 PMC5830977 28988989

[48] Kotni, M. K., Zhao, M. & Wei, D. Q. Gene expression profiles and protein-protein interaction networks in amyotrophic lateral sclerosis patients with C9orf72 mutation. *Orphanet J. Rare Dis.* **11**, 148 (2016). 27814735 10.1186/s13023-016-0531-y PMC5097384

[49] Ziff, O. J. et al. Integrated transcriptome landscape of ALS identifies genome instability linked to TDP-43 pathology. *Nat. Commun.* **14**, 2176 (2023). 37080969 10.1038/s41467-023-37630-6 PMC10119258

[50] Šušnjar, U. et al. Cell environment shapes TDP-43 function with implications in neuronal and muscle disease. *Commun. Biol.* **5**, 314 (2022). 35383280 10.1038/s42003-022-03253-8 PMC8983780

[51] Imaizumi, K., Ideno, H., Sato, T., Morimoto, S. & Okano, H. Pathogenic mutation of TDP-43 impairs RNA processing in a cell type-specific manner: implications for the pathogenesis of ALS/FTLD. *eNeuro* **9**, 1–12 (2022). 10.1523/ENEURO.0061-22.2022 PMC9186108 35641224

[52] Ling, S. C. et al. ALS-associated mutations in TDP-43 increase its stability and promote TDP-43 complexes with FUS/TLS. *Proc. Natl. Acad. Sci. U S A*. **107**, 13318–13323 (2010). 20624952 10.1073/pnas.1008227107 PMC2922163

[53] Paz, I., Kosti, I., Ares, M., Cline, M. & Mandel-Gutfreund, Y. RBPmap: a web server for mapping binding sites of RNA-binding proteins. *Nucleic Acids Res.* **42**, W361–W367 (2014). 24829458 10.1093/nar/gku406 PMC4086114

[54] Andrés-León, E., González Peña, D., Gómez-López, G. & Pisano, D. G. miRGate: a curated database of human, mouse and rat miRNA–mRNA targets. *Database*. **2015**, bav035 (2015). 10.1093/database/bav035 PMC4390609 25858286

[55] Peek, S. L., Mah, K. M. & Weiner, J. A. Regulation of neural circuit formation by protocadherins. *Cell. Mol. Life Sci.* **74**, 4133–4157 (2017). 28631008 10.1007/s00018-017-2572-3 PMC5643215

[56] Jonas, S. & Izaurralde, E. Towards a molecular Understanding of microRNA-mediated gene silencing. *Nat. Rev. Genet.* **16** (2015). 10.1038/nrg3965 26077373

[57] Kanehisa, M. & Goto, S. KEGG: Kyoto encyclopedia of genes and genomes. *Nucleic Acids Res.* **28**, 27–30 (2000). 10592173 10.1093/nar/28.1.27 PMC102409

[58] Kanehisa, M., Furumichi, M., Sato, Y., Kawashima, M. & Ishiguro-Watanabe, M. KEGG for taxonomy-based analysis of pathways and genomes. *Nucleic Acids Res.* **51**, D587–D592 (2023). 36300620 10.1093/nar/gkac963 PMC9825424

[59] Paganoni, S. et al. Trial of sodium phenylbutyrate–taurursodiol for amyotrophic lateral sclerosis. *N Engl. J. Med.* **383**, 919–930 (2020). 32877582 10.1056/NEJMoa1916945 PMC9134321

[60] Paganoni, S. et al. Long-term survival of participants in the CENTAUR trial of sodium phenylbutyrate-taurursodiol in amyotrophic lateral sclerosis. *Muscle Nerve*. **63**, 31–39 (2021). 33063909 10.1002/mus.27091 PMC7820979

[61] Paganoni, S. et al. Survival analyses from the CENTAUR trial in amyotrophic lateral sclerosis: evaluating the impact of treatment crossover on outcomes. *Muscle Nerve*. **1–6**10.1002/mus.27569 (2022). 10.1002/mus.27569 PMC9540225 35508892

[62] Rabut, G. & Peter, M. Function and regulation of protein neddylation. *EMBO Rep.* **9**, 969–976 (2008). 18802447 10.1038/embor.2008.183 PMC2572130

[63] Klim, J. R. et al. ALS-implicated protein TDP-43 sustains levels of STMN2, a mediator of motor neuron growth and repair. *Nat. Neurosci.* **22**, 167–179 (2019). 30643292 10.1038/s41593-018-0300-4 PMC7153761

[64] Melamed, Z. et al. Premature polyadenylation-mediated loss of stathmin-2 is a hallmark of TDP-43-dependent neurodegeneration. *Nat. Neurosci.* **22**, 180–190 (2019). 30643298 10.1038/s41593-018-0293-z PMC6348009

[65] Smith, A. S. T. et al. Human induced pluripotent stem cell-derived TDP-43 mutant neurons exhibit consistent functional phenotypes across multiple gene edited lines despite transcriptomic and splicing discrepancies. *Front. Cell. Dev. Biol.* **9**, 1–18 (2021). 10.3389/fcell.2021.728707 PMC8511491 34660586

[66] Mitsuzawa, S. et al. Reduced PHOX2B stability causes axonal growth impairment in motor neurons with TARDBP mutations. *Stem Cell. Rep.* **16**, 1527–1541 (2021). 10.1016/j.stemcr.2021.04.021 PMC8190591 34048688

[67] Yen, Y. P. et al. Dlk1-Dio3 locus-derived LncRNAs perpetuate postmitotic motor neuron cell fate and subtype identity. *Elife* **7**, (2018). 10.7554/eLife.38080 PMC6221546 30311912

[68] Polymenidou, M. et al. Long pre-mRNA depletion and RNA missplicing contribute to neuronal vulnerability from loss of TDP-43. *Nat. Neurosci.* **14**, 459–468 (2011). 21358643 10.1038/nn.2779 PMC3094729

[69] Tollervey, J. R. et al. Characterizing the RNA targets and position-dependent splicing regulation by TDP-43. *Nat. Neurosci.* **14**, 452–458 (2011). 21358640 10.1038/nn.2778 PMC3108889

[70] Shi, C. et al. CHCHD2 gene mutations in familial and sporadic Parkinson’s disease. *Neurobiol. Aging* 38, 217.e9-217.e13 (2016). 10.1016/j.neurobiolaging.2015.10.040 26705026

[71] Funayama, M. et al. CHCHD2 mutations in autosomal dominant late-onset parkinson’s disease: a genome-wide linkage and sequencing study. *Lancet Neurol.* **14**, 274–282 (2015). 25662902 10.1016/S1474-4422(14)70266-2

[72] Ogaki, K. et al. Mitochondrial targeting sequence variants of the CHCHD2 gene are a risk for lewy body disorders. *Neurology* **85**, 2016–2025 (2015). 26561290 10.1212/WNL.0000000000002170 PMC4676755

[73] Bannwarth, S. et al. A mitochondrial origin for frontotemporal dementia and amyotrophic lateral sclerosis through CHCHD10 involvement. *Brain* **137**, 2329–2345 (2014). 24934289 10.1093/brain/awu138 PMC4107737

[74] Chaussenot, A. et al. Screening of CHCHD10 in a French cohort confirms the involvement of this gene in frontotemporal dementia with amyotrophic lateral sclerosis patients. *Neurobiol. Aging*. **35**, 2884e1–2884e4 (2014). 10.1016/j.neurobiolaging.2014.07.022 25155093

[75] Johnson, J. O. et al. Mutations in the CHCHD10 gene are a common cause of Familial amyotrophic lateral sclerosis. *Brain* **137**, e311–e311 (2014). 25261972 10.1093/brain/awu265 PMC4240285

[76] Alves, C. J. et al. Gene expression profiling for human iPS-derived motor neurons from sporadic ALS patients reveals a strong association between mitochondrial functions and neurodegeneration. *Front. Cell. Neurosci.* **9**, 1–25 (2015). 26300727 10.3389/fncel.2015.00289 PMC4523944

[77] Bean, D. M., Al-Chalabi, A., Dobson, R. J. B. & Iacoangeli, A. A knowledge-based machine learning approach to gene prioritisation in amyotrophic lateral sclerosis. *Genes (Basel)*. **11**, 1–17 (2020). 10.3390/genes11060668 PMC7349022 32575372

[78] Held, A. et al. iPSC motor neurons, but not other derived cell types, capture gene expression changes in postmortem sporadic ALS motor neurons. *Cell. Rep.* **42**, 113046 (2023). 37651231 10.1016/j.celrep.2023.113046 PMC10622181

[79] Ho, R. et al. ALS disrupts spinal motor neuron maturation and aging pathways within gene co-expression networks. *Nat. Neurosci.* **19**, 1256–1267 (2016). 27428653 10.1038/nn.4345 PMC5003654

[80] Gatto, N. et al. Directly converted astrocytes retain the ageing features of the donor fibroblasts and elucidate the astrocytic contribution to human CNS health and disease. *Aging Cell.* **20**, e13281 (2021). 33314575 10.1111/acel.13281 PMC7811849

[81] Coyne, A. N. et al. Nuclear accumulation of CHMP7 initiates nuclear pore complex injury and subsequent TDP-43 dysfunction in sporadic and familial ALS. *Sci. Transl Med.* **13**, 1–14 (2021). 10.1126/scitranslmed.abe1923 PMC9022198 34321318

[82] Rothstein, J. D. et al. G2C4 targeting antisense oligonucleotides potently mitigate TDP-43 dysfunction in human C9orf72 ALS/FTD induced pluripotent stem cell derived neurons. *Acta Neuropathol.* **147**, 1 (2024). 10.1007/s00401-023-02652-3 PMC10840905 38019311

[83] De Felice, B. et al. Wide-ranging analysis of MicroRNA profiles in sporadic amyotrophic lateral sclerosis using next-generation sequencing. *Front. Genet.* **9**, (2018). 10.3389/fgene.2018.00310 PMC6102490 30154826

[84] Wakabayashi, K. et al. Analysis of MicroRNA from archived formalin-fixed paraffin-embedded specimens of amyotrophic lateral sclerosis. *Acta Neuropathol. Commun.* **2**, (2014). 10.1186/s40478-014-0173-z PMC4279903 25497327

[85] Figueroa-Romero, C. et al. Expression of MicroRNAs in human post-mortem amyotrophic lateral sclerosis spinal cords provides insight into disease mechanisms. *Mol Cell. Neurosci.* **71**, (2016). 10.1016/j.mcn.2015.12.008 PMC4761498 26704906

[86] Matamala, J. M. et al. Genome-wide Circulating MicroRNA expression profiling reveals potential biomarkers for amyotrophic lateral sclerosis. *Neurobiol. Aging*. **64**, (2018). 10.1016/j.neurobiolaging.2017.12.020 29458840

[87] Taguchi, Y. H. & Wang, H. Exploring MicroRNA biomarker for amyotrophic lateral sclerosis. *Int. J. Mol. Sci.* **19**, (2018). 10.3390/ijms19051318 PMC5983737 29710810

[88] Si, Y. et al. Muscle MicroRNA signatures as biomarkers of disease progression in amyotrophic lateral sclerosis. *Neurobiol. Dis.* **114**, (2018). 10.1016/j.nbd.2018.02.009 PMC5891369 29486297

[89] Katsu, M. et al. MicroRNA expression profiles of neuron-derived extracellular vesicles in plasma from patients with amyotrophic lateral sclerosis. *Neurosci. Lett.* **708**, (2019). 10.1016/j.neulet.2019.03.048 31173847

[90] Capauto, D. et al. A regulatory circuitry between Gria2, miR-409, and miR-495 is affected by ALS FUS mutation in ESC-derived motor neurons. *Mol. Neurobiol.* **55**, 7635–7651 (2018). 29430619 10.1007/s12035-018-0884-4 PMC6132778

[91] Luigetti, M. et al. A novel compound heterozygous ALS2 mutation in two Italian siblings with juvenile amyotrophic lateral sclerosis. *Amyotroph. Lateral Scler. Front. Degener*. **14**, 470–472 (2013). 10.3109/21678421.2012.756036 23282280

[92] Sheerin, U. M. et al. ALS2 mutations: juvenile amyotrophic lateral sclerosis and generalized dystonia. *Neurology* **82**, 1065–1067 (2014). 24562058 10.1212/WNL.0000000000000254 PMC3962990

[93] Gilley, J. et al. Enrichment of SARM1 alleles encoding variants with constitutively hyperactive NADase in patients with ALS and other motor nerve disorders. *Elife* **10**, e70905 (2021). 34796871 10.7554/eLife.70905 PMC8735862

[94] van Rheenen, W. et al. Genome-wide association analyses identify new risk variants and the genetic architecture of amyotrophic lateral sclerosis. *Nat. Genet.* **48**, 1043–1048 (2016). 27455348 10.1038/ng.3622 PMC5556360

[95] Wang, Y., Luo, J., Zhang, H. & Lu, J. MicroRNAs in the same clusters evolve to coordinately regulate functionally related genes. *Mol. Biol. Evol.* **33**, 2232–2247 (2016). 27189568 10.1093/molbev/msw089 PMC4989102

[96] Winter, J. MicroRNAs of the miR379–410 cluster: new players in embryonic neurogenesis and regulators of neuronal function. *Neurogenesis* **2**, e1004970 (2015). 27504472 10.1080/23262133.2015.1004970 PMC4973610

[97] Marty, V. & Cavaillé, J. Imprinted small noncoding RNA genes in brain function and behaviour. *Curr. Opin. Behav. Sci.* **25**, 8–14 (2019).

[98] Jimenez-Mateos, E. M. et al. Silencing microRNA-134 produces neuroprotective and prolonged seizure-suppressive effects. *Nat. Med.* **18**, 1087–1094 (2012). 22683779 10.1038/nm.2834 PMC3438344

[99] Wang, X. M., Jia, R. H., Wei, D., Cui, W. Y. & Jiang, W. MiR-134 blockade prevents status epilepticus like-activity and is neuroprotective in cultured hippocampal neurons. *Neurosci. Lett.* **572**, 20–25 (2014). 24810882 10.1016/j.neulet.2014.04.049

[100] Santarelli, D. M., Beveridge, N. J., Tooney, P. A. & Cairns, M. J. Upregulation of dicer and microRNA expression in the dorsolateral prefrontal cortex brodmann area 46 in schizophrenia. *Biol. Psychiatry*. **69**, 180–187 (2011). 21111402 10.1016/j.biopsych.2010.09.030

[101] Gardiner, E. et al. Imprinted DLK1-DIO3 region of 14q32 defines a schizophrenia-associated MiRNA signature in peripheral blood mononuclear cells. *Mol. Psychiatry*. **17**, 827–840 (2012). 21727898 10.1038/mp.2011.78 PMC3404364

[102] Sarachana, T., Zhou, R., Chen, G., Manji, H. K. & Hu, V. W. Investigation of post-transcriptional gene regulatory networks associated with autism spectrum disorders by microRNA expression profiling of lymphoblastoid cell lines. *Genome Med.* **2**, 23 (2010). 20374639 10.1186/gm144 PMC2873801

[103] Wu, H. et al. Genome-wide analysis reveals methyl-CpG-binding protein 2-dependent regulation of microRNAs in a mouse model of Rett syndrome. *Proc. Natl. Acad. Sci. U S A*. **107**, 18161–18166 (2010). 20921386 10.1073/pnas.1005595107 PMC2964235

[104] Henriksen, M., Johnsen, K. B., Olesen, P., Pilgaard, L. & Duroux, M. MicroRNA expression signatures and their correlation with clinicopathological features in glioblastoma multiforme. *NeuroMolecular Med.* **16**, 565–577 (2014). 24817689 10.1007/s12017-014-8309-7

[105] Gattolliat, C. H. et al. Expression of miR-487b and miR-410 encoded by 14q32.31 locus is a prognostic marker in neuroblastoma. *Br. J. Cancer*. **105**, 1352–1361 (2011). 21970883 10.1038/bjc.2011.388 PMC3241557

[106] Tsang, E. K. et al. Small RNA sequencing in cells and exosomes identifies eQTLs and 14q32 as a region of active export. *G3 (Bethesda)*. **7**, 31–39 (2017). 27799337 10.1534/g3.116.036137 PMC5217120

[107] Valbuena, G. N. et al. The 14q32 maternally imprinted locus is a major source of longitudinally stable circulating MicroRNAs as measured by small RNA sequencing. *Sci. Rep.* **9**, 15787 (2019). 31673048 10.1038/s41598-019-51948-6 PMC6823392

[108] Lo, T. W. et al. Extracellular vesicles in serum and central nervous system tissues contain MicroRNA signatures in sporadic amyotrophic lateral sclerosis. *Front. Mol. Neurosci.* **14**, (2021). 10.3389/fnmol.2021.739016 PMC8586523 34776863

[109] Saucier, D. et al. Identification of a circulating MiRNA signature in extracellular vesicles collected from amyotrophic lateral sclerosis patients. *Brain Res.* **1708**, 100–108 (2019). 30552897 10.1016/j.brainres.2018.12.016

[110] Rupaimoole, R. & Slack, F. J. MicroRNA therapeutics: towards a new era for the management of cancer and other diseases. *Nat. Rev. Drug Discov*. **16**, 203–222 (2017). 28209991 10.1038/nrd.2016.246

[111] Bhaskaran, V., Yao, Y., Bei, F. & Peruzzi, P. Engineering, delivery, and biological validation of artificial microRNA clusters for gene therapy applications. *Nat. Protoc.* **14**, 3538–3553 (2019). 31748752 10.1038/s41596-019-0241-8 PMC7089775

[112] Diener, C., Keller, A. & Meese, E. Emerging concepts of MiRNA therapeutics: from cells to clinic. *Trends Genet.* **38**, 613–626 (2022). 35303998 10.1016/j.tig.2022.02.006

[113] Theodoris, C. V. et al. Network-based screen in iPSC-derived cells reveals therapeutic candidate for heart valve disease. *Sci. (80-)*. 10.1126/science.abd0724 (2021). 10.1126/science.abd0724 PMC7880903 33303684

[114] Theodoris, C. V. et al. Human disease modeling reveals integrated transcriptional and epigenetic mechanisms of NOTCH1 haploinsufficiency. *Cell* **160**, 1072–1086 (2015). 25768904 10.1016/j.cell.2015.02.035 PMC4359747

[115] Zhu, J. et al. Prediction of drug efficacy from transcriptional profiles with deep learning. *Nat. Biotechnol.* **39**, 1444–1452 (2021). 34140681 10.1038/s41587-021-00946-z

[116] Delahaye-Duriez, A. et al. Rare and common epilepsies converge on a shared gene regulatory network providing opportunities for novel antiepileptic drug discovery. *Genome Biol.* **17**, 1–18 (2016). 27955713 10.1186/s13059-016-1097-7 PMC5154105

[117] Srivastava, P. K. et al. A systems-level framework for drug discovery identifies Csf1R as an anti-epileptic drug target. *Nat. Commun.* **9**, 3561 (2018). 30177815 10.1038/s41467-018-06008-4 PMC6120885

[118] Brueggeman, L. et al. Drug repositioning in epilepsy reveals novel Antiseizure candidates. *Ann. Clin. Transl Neurol.* **6**, 295–309 (2019). 30847362 10.1002/acn3.703 PMC6389756

[119] Readhead, B. et al. Expression-based drug screening of neural progenitor cells from individuals with schizophrenia. *Nat. Commun.* **9**, (2018). 10.1038/s41467-018-06515-4 PMC6200740 30356048

[120] Swarup, V. et al. Identification of evolutionarily conserved gene networks mediating neurodegenerative dementia. *Nat. Med.* **25**, 152–164 (2019). 30510257 10.1038/s41591-018-0223-3 PMC6602064

[121] Patten, S. A. et al. Neuroleptics as therapeutic compounds stabilizing neuromuscular transmission in amyotrophic lateral sclerosis. *JCI Insight*. **2**, e97152 (2017). 29202456 10.1172/jci.insight.97152 PMC5752378

[122] Yue, W. et al. Inhibition of the MEK / ERK pathway suppresses immune overactivation and mitigates TDP – 43 toxicity in a Drosophila model of ALS. *Immun. Ageing*. **1–13**10.1186/s12979-023-00354-8 (2023). 10.1186/s12979-023-00354-8 PMC10280928 37340309

[123] Ayala, V. et al. Cell stress induces TDP-43 pathological changes associated with ERK1/2 dysfunction: implications in ALS. *Acta Neuropathol.* **122**, 259–270 (2011). 21706176 10.1007/s00401-011-0850-y

[124] Chung, Y. H. et al. Immunohistochemical study on the distribution of phosphorylated extracellular signal-regulated kinase (ERK) in the central nervous system of SOD1G93A Transgenic mice. *Brain Res.* **1050**, 203–209 (2005). 15978558 10.1016/j.brainres.2005.05.060

[125] Howell, S. J. et al. Retinal inflammation, oxidative stress, and vascular impairment is ablated in diabetic mice receiving XMD8-92 treatment. *Front. Pharmacol.* **12**, 732630 (2021). 34456740 10.3389/fphar.2021.732630 PMC8385489

[126] Zhang, S., Fujita, Y., Matsuzaki, R. & Yamashita, T. Class I histone deacetylase (HDAC) inhibitor CI-994 promotes functional recovery following spinal cord injury. *Cell. Death Dis.* **9**, 460 (2018). 29700327 10.1038/s41419-018-0543-8 PMC5919919

[127] Sada, N. et al. Inhibition of HDAC increases BDNF expression and promotes neuronal rewiring and functional recovery after brain injury. *Cell. Death Dis.* **11**, 655 (2020). 32811822 10.1038/s41419-020-02897-w PMC7434917

[128] Marinho, D. et al. Reduction of class I histone deacetylases ameliorates ER-mitochondria cross-talk in Alzheimer’s disease. *Aging Cell.* **22**, e13895 (2023). 37358017 10.1111/acel.13895 PMC10410063

[129] Xiao, Z. & Vijayalakshmi, A. Protective effect of piperlongumine on inflammation and oxidative stress against ischemia-reperfusion injury in animal kidney. *Bratisl Lek Listy*. **123**, 878–884 (2022). 36342874 10.4149/BLL_2022_140

[130] Liu, J. et al. Piperlongumine restores the balance of autophagy and apoptosis by increasing BCL2 phosphorylation in rotenone-induced Parkinson disease models. *Autophagy* **14**, 845–861 (2018). 29433359 10.1080/15548627.2017.1390636 PMC6070010

[131] Yang, G., Ham, I. & Choi, H. Y. Anti-inflammatory effect of prunetin via the suppression of NF-κB pathway. *Food Chem. Toxicol.* **58**, 124–132 (2013). 23597450 10.1016/j.fct.2013.03.039

[132] Scudder, S. L. & Patrick, G. N. Synaptic structure and function are altered by the neddylation inhibitor MLN4924. *Mol. Cell. Neurosci.* **65**, 52–57 (2015). 25701678 10.1016/j.mcn.2015.02.010 PMC4844066

[133] Vogl, A. M. et al. Neddylation inhibition impairs spine development, destabilizes synapses and deteriorates cognition. *Nat. Neurosci.* **18**, 239–251 (2015). 25581363 10.1038/nn.3912

[134] Li, L. et al. Enzymatic activity of the scaffold protein rapsyn for synapse formation. *Neuron* **92**, 1007–1019 (2016). 27839998 10.1016/j.neuron.2016.10.023 PMC5366040

[135] Brockmann, M. M. et al. Neddylation regulates excitatory synaptic transmission and plasticity. *Sci. Rep.* **9**, 17935 (2019). 31784571 10.1038/s41598-019-54182-2 PMC6884593

[136] Vogl, A. M. et al. Global site-specific neddylation profiling reveals that neddylated cofilin regulates actin dynamics. *Nat. Struct. Mol. Biol.* **27**, 210–220 (2020). 32015554 10.1038/s41594-019-0370-3

[137] Chen, Y. Z. APP induces neuronal apoptosis through APP-BP1-mediated downregulation of β-catenin. *Apoptosis* **9**, 415–422 (2004). 15192323 10.1023/B:APPT.0000031447.05354.9f

[138] Hua, W. et al. Suppression of glioblastoma by targeting the overactivated protein neddylation pathway. *Neuro Oncol.* **17**, 1333–1343 (2015). 25904638 10.1093/neuonc/nov066 PMC4578582

[139] Brandt, B. et al. A promising way to overcome temozolomide resistance through Inhibition of protein neddylation in glioblastoma cell lines. *Int. J. Mol. Sci.* **24**, 7929 (2023). 37175636 10.3390/ijms24097929 PMC10178391

[140] Mori, F. et al. Accumulation of NEDD8 in neuronal and glial inclusions of neurodegenerative disorders. *Neuropathol. Appl. Neurobiol.* **31**, 53–61 (2005). 15634231 10.1111/j.1365-2990.2004.00603.x

[141] Kim, A. Y. et al. SCCRO (DCUN1D1) is an essential component of the E3 complex for neddylation. *J. Biol. Chem.* **283**, 33211–33220 (2008). 18826954 10.1074/jbc.M804440200 PMC2586271

[142] Villa, C. et al. DCUN1D1 is a risk factor for frontotemporal Lobar degeneration. *Eur. J. Neurol.* **16**, 870–873 (2009). 19473369 10.1111/j.1468-1331.2009.02611.x

[143] Fu, D. J. & Wang, T. Targeting NEDD8-activating enzyme for cancer therapy: developments, clinical trials, challenges and future research directions. *J. Hematol. Oncol.* **16**, 87 (2023). 37525282 10.1186/s13045-023-01485-7 PMC10388525

[144] Yu, H. et al. The NEDD8-activating enzyme inhibitor MLN4924 reduces ischemic brain injury in mice. *Proc. Natl. Acad. Sci. U. S. A.* **119**, (2022). 10.1073/pnas.2111896119 PMC8833173 35101976

[145] Andérica-Romero, A. C., Hernández-Damián, J., Vázquez-Cervantes, G. I., Torres, I. & Pedraza-Chaverri, J. The MLN4924 inhibitor exerts a neuroprotective effect against oxidative stress injury via Nrf2 protein accumulation. *Redox Biol.* **8**, 341–347 (2016). 26966893 10.1016/j.redox.2016.02.008 PMC4789348

[146] Kassouf, T. et al. Targeting the NEDP1 enzyme to ameliorate ALS phenotypes through stress granule disassembly. *Sci. Adv.* **9**, (2023). 10.1126/sciadv.abq7585 PMC10065448 37000881

[147] Hofweber, M. & Dormann, D. Friend or foe—Post-translational modifications as regulators of phase separation and RNP granule dynamics. *J. Biol. Chem.* **294**, 7137–7150 (2019). 30587571 10.1074/jbc.TM118.001189 PMC6509508

[148] Jayabalan, A. K. et al. NEDDylation promotes stress granule assembly. *Nat. Commun.* **7**, 12125 (2016). 27381497 10.1038/ncomms12125 PMC4935812

[149] Markmiller, S. et al. Active protein neddylation or ubiquitylation is dispensable for stress granule dynamics. *Cell. Rep.* **27**, 1356–1363e3 (2019). 31042464 10.1016/j.celrep.2019.04.015 PMC6508666

[150] Maghames, C. M. et al. NEDDylation promotes nuclear protein aggregation and protects the ubiquitin proteasome system upon proteotoxic stress. *Nat. Commun.* **9**, 4376 (2018). 30349034 10.1038/s41467-018-06365-0 PMC6197266

[151] Chen, C. X. Q. et al. A multistep workflow to evaluate newly generated iPSCs and their ability to generate different cell types. *Methods Protoc.* **4**, 50 (2021). 34287353 10.3390/mps4030050 PMC8293472

[152] Deneault, E. et al. A streamlined CRISPR workflow to introduce mutations and generate isogenic iPSCs for modeling amyotrophic lateral sclerosis. *Methods* **203**, 297–310 (2022). 34500068 10.1016/j.ymeth.2021.09.002

[153] Bourgey, M. et al. GenPipes: an open-source framework for distributed and scalable genomic analyses. *Gigascience* **8**, 1–11 (2019). 10.1093/gigascience/giz037 PMC6559338 31185495

[154] Bolger, A. M., Lohse, M. & Usadel, B. Trimmomatic: A flexible trimmer for illumina sequence data. *Bioinformatics* **30**, (2014). 10.1093/bioinformatics/btu170 PMC4103590 24695404

[155] Dobin, A. et al. STAR: ultrafast universal RNA-seq aligner. *Bioinformatics* **29**, 15–21 (2013). 23104886 10.1093/bioinformatics/bts635 PMC3530905

[156] Trapnell, C. et al. Transcript assembly and quantification by RNA-Seq reveals unannotated transcripts and isoform switching during cell differentiation. *Nat. Biotechnol.* **28**, 511–515 (2010). 20436464 10.1038/nbt.1621 PMC3146043

[157] Love, M. I., Huber, W. & Anders, S. Moderated estimation of fold change and dispersion for RNA-seq data with DESeq2. *Genome Biol.* **15**, 550 (2014). 25516281 10.1186/s13059-014-0550-8 PMC4302049

[158] Potla, P., Ali, S. A. & Kapoor, M. A bioinformatics approach to microRNA-sequencing analysis. *Osteoarthr. Cartil. Open.* **3**, 100131 (2021). 36475076 10.1016/j.ocarto.2020.100131 PMC9718162

[159] Langmead, B., Trapnell, C., Pop, M. & Salzberg, S. L. Ultrafast and memory-efficient alignment of short DNA sequences to the human genome. *Genome Biol.* **10**, R25 (2009). 19261174 10.1186/gb-2009-10-3-r25 PMC2690996

[160] Griffiths-Jones, S., Grocock, R. J., van Dongen, S., Bateman, A. & Enright, A. J. MiRBase: MicroRNA sequences, targets and gene nomenclature. *Nucleic Acids Res.* **34**, D140–D144 (2006). 16381832 10.1093/nar/gkj112 PMC1347474

[161] Kozomara, A., Birgaoanu, M. & Griffiths-Jones, S. MiRBase: from MicroRNA sequences to function. *Nucleic Acids Res.* **47**, D155–D162 (2019). 30423142 10.1093/nar/gky1141 PMC6323917

[162] Kozomara, A. & Griffiths-Jones, S. MiRBase: annotating high confidence MicroRNAs using deep sequencing data. *Nucleic Acids Res.* **42**, D68–73 (2014). 24275495 10.1093/nar/gkt1181 PMC3965103

[163] Kozomara, A. & Griffiths-Jones, S. MiRBase: integrating MicroRNA annotation and deep-sequencing data. *Nucleic Acids Res.* **39**, D152–D157 (2011). 21037258 10.1093/nar/gkq1027 PMC3013655

[164] Griffiths-Jones, S., Saini, H. K., van Dongen, S. & Enright, A. J. MiRBase: tools for microRNA genomics. *Nucleic Acids Res.* **36**, D154–D158 (2007). 17991681 10.1093/nar/gkm952 PMC2238936

[165] Danecek, P. et al. Twelve years of SAMtools and BCFtools. *Gigascience* **10**, (2021). 10.1093/gigascience/giab008 PMC7931819 33590861

[166] Quinlan, A. R. & Hall, I. M. BEDTools: a flexible suite of utilities for comparing genomic features. *Bioinformatics* **26**, 841–842 (2010). 20110278 10.1093/bioinformatics/btq033 PMC2832824

[167] Cox, D. R. & Reid, N. Parameter orthogonality and approximate conditional inference. *J. R Stat. Soc. Ser. B*. **49**, 1–18 (1987).

[168] Peltier, H. J. & Latham, G. J. Normalization of MicroRNA expression levels in quantitative RT-PCR assays: identification of suitable reference RNA targets in normal and cancerous human solid tissues. *RNA* **14**, 844–852 (2008). 18375788 10.1261/rna.939908 PMC2327352

[169] Castellanos-Montiel, M. J. et al. An optimized workflow to generate and characterize iPSC-derived motor neuron (MN) spheroids. *Cells* **12**, 545 (2023). 36831212 10.3390/cells12040545 PMC9954647

