# Supplementary material for: Transcriptome-based screening in TARDBP/TDP-43 knock-in motor neurons identifies the NEDD8-activating enzyme inhibitor MLN4924
Source: Sci Rep. 2025 Aug 5;15:28555. doi: 10.1038/s41598-025-12147-8 (PMC12325802; doi:10.1038/s41598-025-12147-8)
Supplement: Supplementary file 6 — Supplementary Material 6 [file 41598_2025_12147_MOESM6_ESM.pdf]

Transcriptome-based screening in TARDBP/TDP-43 knock-in motor neurons identifies the NEDD8-activating enzyme inhibitor MLN4924

Sarah Lépine[1,2,3], Gilles Maussion[1], Alexandria Schneider[1], Angela Nauleau-Javaudin[1,4], María José Castellanos-Montiel[1,2], Georgina Jiménez Ambriz[5], Dan Spiegelman[5], Narges Abdian[1], Anna Krystina Franco-Flores[1], Ghazal Haghi[1], Lale Gursu[1], Michael R. Fiorini[5,6], Allison A. Dilliott[2,5], Sali M. K. Farhan[2,5,6], Mathilde Chaineau[1,*], and Thomas M. Durcan[1,2,*]

[1]Early Drug Discovery Unit (EDDU), The Neuro-Montreal Neurological Institute and Hospital, McGill University, Montreal, Quebec, Canada H3A 1A1
[2]Department of Neurology and Neurosurgery, McGill University, Montreal, Quebec, Canada H3A 1A1
[3]Faculty of Medicine and Health Sciences, McGill University, Montreal, Quebec, Canada H3G 2M1
[4]Faculté de médecine, Université de Montréal, Montreal, Quebec, Canada H3T 1J4
[5]The Neuro Bioinformatics Core Facility, The Neuro-Montreal Neurological Institute and Hospital, McGill University, Montreal, Quebec, Canada H3A 1A1
[6]Department of Human Genetics, McGill University, Montreal, Quebec, Canada H3A0G4

*Corresponding authors: thomas.durcan@mcgill.ca (T.M.D.), mathilde.chaineau@mcgill.ca (M.C.)

Supplementary Figures

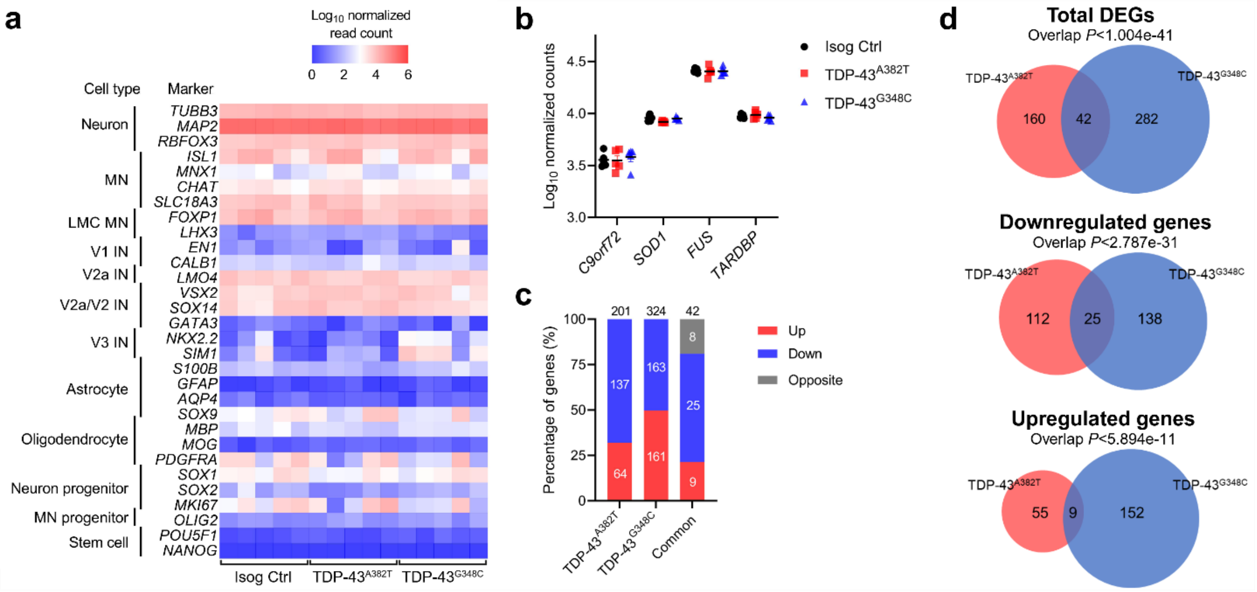

Supplementary Fig. S1 Transcriptomic profiling of TDP-43^{A382T} and TDP-43^{G348C} MNs. a Heatmap of normalized counts of cell type markers determined by RNA-seq. **b** Normalized counts of ALS-associated genes. **c, d** Stacked bar graph (**c**) and Venn diagram (**d**) comparing differentially expressed genes (DEGs) in TDP-43 MNs relative to isogenic control. n=5. **e** Venn diagram comparing DEGs and a published list of known TDP-43 mRNAs binding targets.

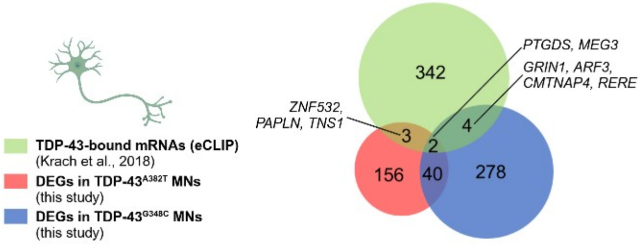

Supplementary Fig. S2 Few dysregulated genes are known TDP-43 targets. Venn diagram comparing differentially expressed genes (DEGs) in TDP-43 MNs and a list of known TDP-43 mRNA bindings targets revealed by enhanced crosslinking and immunoprecipitation (eCLIP) followed by RNA-seq in motor cortex specimen from sporadic ALS patients and healthy individuals[1].

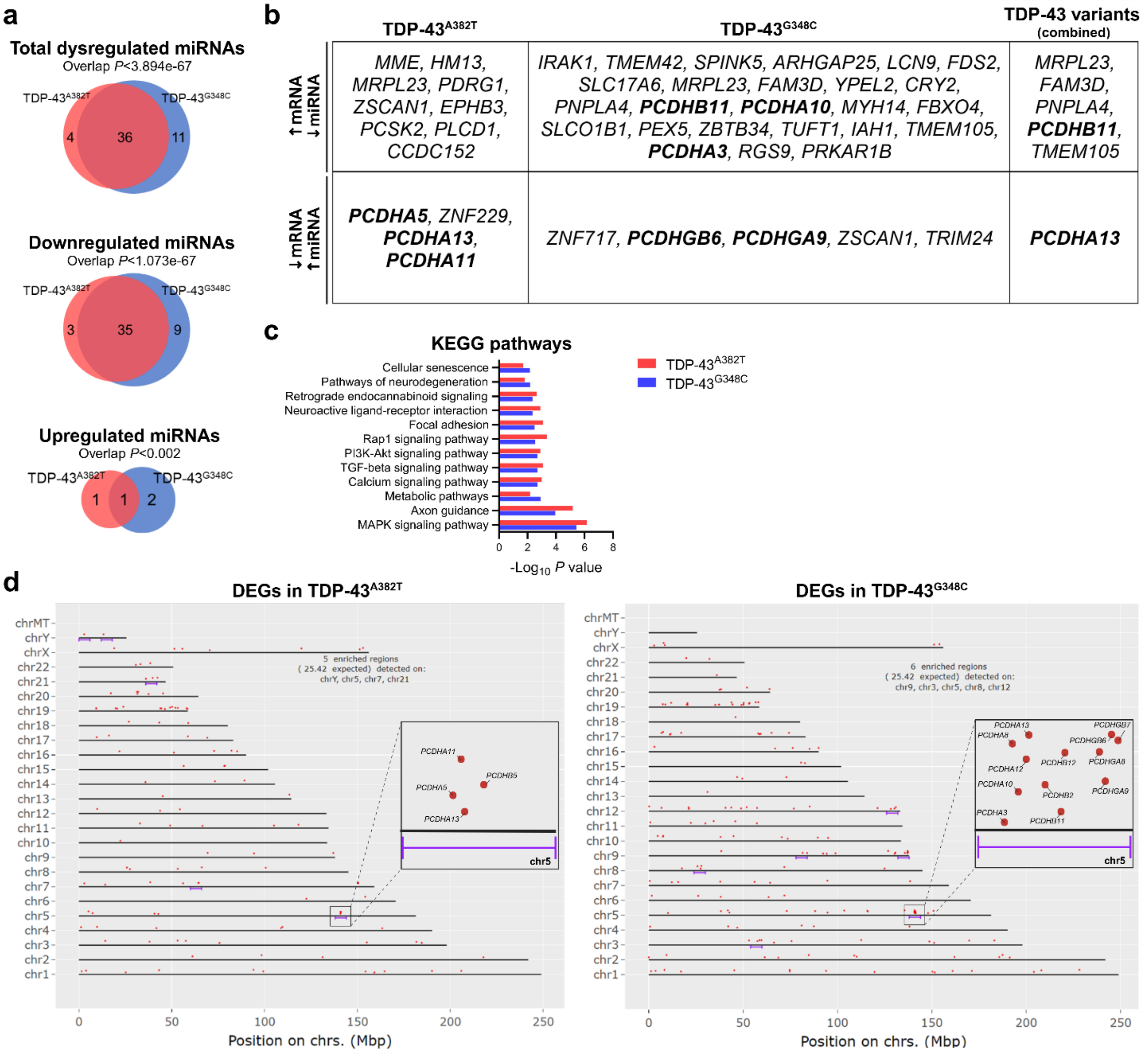

Supplementary Fig. S3 miRNAs profiling and integrated miRNA/mRNA analysis highlight protocadherin-coding genes. a Venn diagram comparing differentially expressed miRNAs in TDP-43 MNs relative to isogenic control. n=5. **b** Differentially expressed genes (DEGs) predicted to be targeted by dysregulated miRNAs. **c** KEGG pathway analysis of predicted target genes of dysregulated miRNAs. **d** Genomic regions of statistical DEGs enrichment (FDR<0.05) determined by the ShinyGO web app. DEGs are enriched at the clustered protocadherin gene locus (chr5q31).

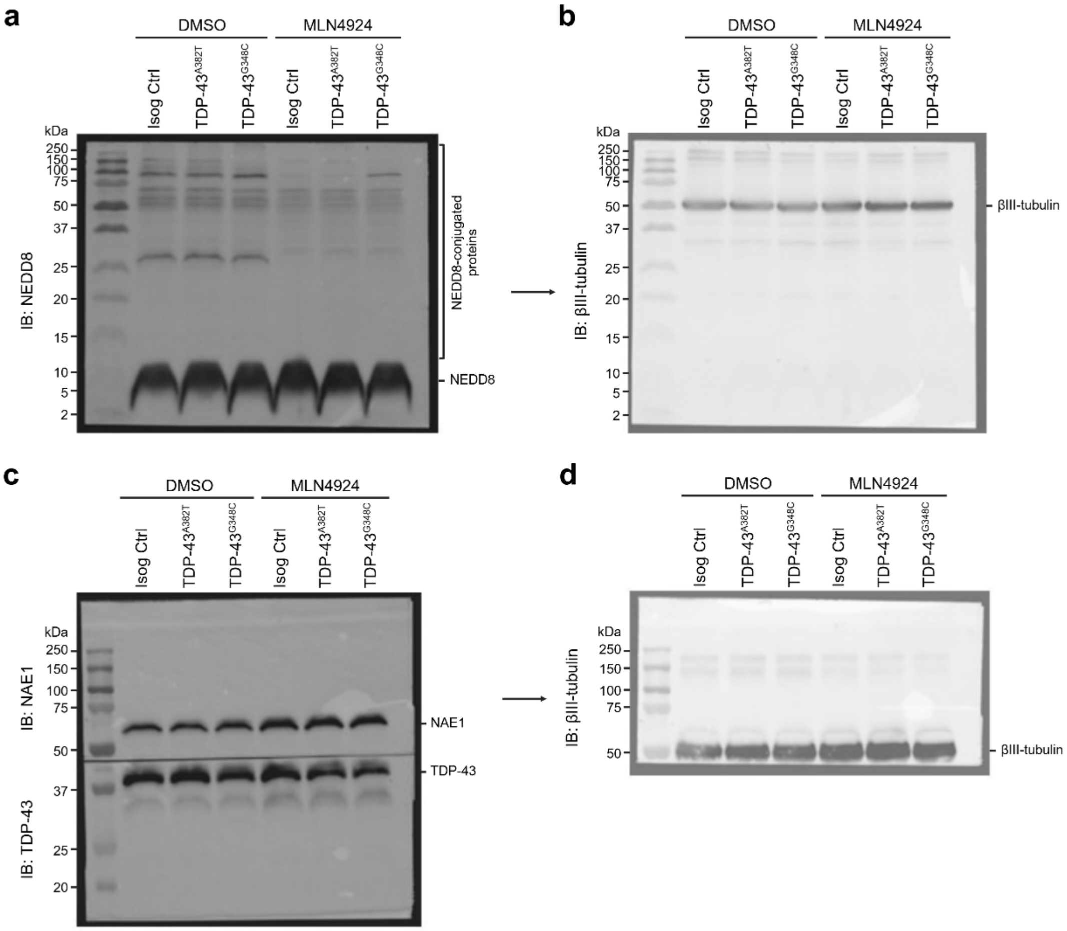

Supplementary Fig. S4 Western blot analysis of NEDDylation proteins and TDP-43 with and without MLN4924 treatment. a-d Uncropped immunoblots from Fig. 6 showing levels of free NEDD8 and NEDD8-conjugated proteins (**a**), NAE1 (**c**), and TDP-43 (**c**) in 2-week MNs treated with DMSO or MLN4924 (1.0 µM). βIII-tubulin was used as loading control (**b,d**). n=3 independent experiments.

Supplementary Tables

Supplementary Table S1. Overview of iPSC lines used

Cell line ID	ALS mutation	Sex	Age	Ethnicity	Primary cell line	Reprogramming method	Reference
AIW002-02	None	Male	37	Caucasian	PBMC	Sendai virus	Chen et al., 2021[2]
TARDBP A382T/AIW002-02	p.A382T	Male	37	Caucasian	Knock-in	n/a	Lépine et al., 2024[3]
TARDBP G348C/AIW002-02	p.G348C	Male	37	Caucasian	Knock-in	n/a	Lépine et al., 2024[3]

* PBMC, peripheral blood mononuclear cell; n/a, not applicable.

Supplementary Table S2. List of primers and TaqMan probes

Gene/miRNA	Reference
18S (RN18S1)	Hs.PT.39a.22214856.g
HPRT1	Hs.PT.58v.45621572
PPIA	Hs.PT.58v.38887593.g
POLR2A	Hs.PT.39a.19639531
ZNF502	Hs.PT.58.24576237
MEG3	Hs.PT.58.25426100
PTGDS	Hs.PT.58.2258847
ZNF283	Hs.PT.58.27732356
RAD51C	Hs.PT.58.40910176
PCDHA13	Hs.PT.58.27085852.g
GPR50	Hs.PT.58.39777811
NNAT	Hs.PT.58.24260117.g
CHCHD2	Hs.PT.58.38445645
PDE12	Hs.PT.58.19439493
CBR1	Hs.PT.56a.22365466
GSE1	Hs.PT.58.40478286
EBF2	Hs.PT.58.818465
PRKCD	Hs.PT.58.21209513
CHRNA2	Hs.PT.58.20688322
NEDD8	Hs01921826_g1
UBA3	Hs01091470_m1
NAE1	Hs01000370_m1
TARDBP	Hs00606522_m1
hsa-miR-191-5p	477952_mir
hsa-miR-432-5p	478101_mir
hsa-miR-654-5p	478368_mir
hsa-miR-381-3p	477816_mir
hsa-miR-485-5p	478126_mir
hsa-miR-411-5p	478086_mir
hsa-miR-127-5p	477889_mir
hsa-miR-370-3p	478326_mir

Supplementary Table S3. Shared differential expressed genes

Gene symbol**	Gene name	Functions/Comments***	LogFC	
			p.A382T	p.G348C
Upregulated				
CYCSP49	CYCS pseudogene 49	Pseudogene	2.925	2.149
PPP1R12BP1	Protein phosphatase 1 regulatory subunit 12B pseudogene 1	Pseudogene	2.743	2.291
MRPL23	Mitochondrial ribosomal protein L23	Mitochondrial translation and metabolism of proteins	1.417	1.324
GSE1	Gse1 coiled-coil protein	May be subunit of a BRAF35-HDAC (BHC) histone deacetylase complex; overexpressed in breast cancer	0.401	0.428
ANKRD36P1	Ankyrin repeat domain 36 pseudogene 1	Pseudogene	2.990	2.644
EBF2	EBF transcription factor 2	Transcription factor	1.897	1.186
NUTM2F	NUT family member 2F	Protein-coding gene of unknown function	0.852	1.084
PRKCD	Protein kinase C delta	Related to autophagy and apoptosis; associated with various human diseases; predicted ALS gene[4]	1.237	0.922
CHRNA2	Cholinergic receptor nicotinic alpha 2 subunit	Nicotinic acetylcholine receptor subunit widely expressed in the brain; associated with epilepsy	1.899	1.637
Downregulated				
ZNF626	Zinc finger protein 626	Predicted to enable DNA-binding transcription factor activity	-0.949	-0.925
ZNF502	Zinc finger protein 502	Predicted to enable DNA-binding transcription factor activity	-7.415	-7.231
LINC00839	Long intergenic non-protein coding RNA 839	lncRNA	-8.769	-8.667
ENSG00000285876	Novel transcript	lncRNA	-11.862	-6.14
ENSG00000269993	Novel transcript	lncRNA	-3.728	-3.179
MEG3	Maternally expressed 3	lncRNA associated with rare syndromes; known TDP-43 target[1,5,6]	-8.439	-9.004
PTGDS	Prostaglandin D2 synthase	Functions as a neuromodulator and trophic factor in the CNS; known TDP-43 target[1]	-3.579	-3.477
ZNF717	Zinc finger protein 717	Kruppel-associated box (KRAB) zinc-finger protein; transcriptional regulator	-7.149	-9.269
LINC02506	Long intergenic non-protein coding RNA 2506	lncRNA	-6.348	-4.170
ZNF283	Zinc finger protein 283	Predicted to enable DNA-binding transcription factor activity	-3.759	-3.404
SVIL-AS1	SVIL antisense RNA 1	lncRNA	-7.275	-7.288
RAD51C	RAD51 paralog C	Homologous recombination and DNA repair	-0.455	-0.301
PCDHA13	Protocadherin alpha 13	Neural cadherin-like cell adhesion protein; enriched in the brain	-6.492	-6.244
ZNF736	Zinc finger protein 736	Predicted to enable DNA-binding transcription factor activity	-5.283	-6.767
ENSG00000229370	Novel transcript	lncRNA	-8.090	-6.443
ENSG00000287069	Novel transcript, antisense to *KCNH8*	lncRNA	-4.777	-4.134
NNAT	Neuronatin	May play a role in the formation and maintenance of the CNS, and regulation of ion channels during brain development	-10.147	-11.453
GPR50	G protein-coupled receptor 50	G-coupled receptor activity; associated with bipolar affective disorder and major depressive disorder	-3.561	-3.455
CHCHD2	Coiled-coil-helix-coiled-coil-helix domain containing 2	Involved in mitochondrion organization; negative regulator of mitochondria-mediated apoptosis; associated with PD; predicted ALS gene[4]	-10.399	-11.938

LINC02527	Long intergenic non-protein coding RNA 2527	lncRNA	-0.873	-1.342
MIR4458HG	MIR4458 host gene	lncRNA	-10.030	-9.931
CCNYL2	Cyclin Y like 2	Predicted to enable cyclin-dependent protein serine/threonine kinase regulator activity	-4.539	-4.344
PDE12	Phosphodiesterase 12	Localized to the mitochondrial matrix; involved in RNA metabolic process, cytokine signaling and mitochondrial mRNA stability	-0.392	-0.393
CBR1	Carbonyl reductase 1	NADPH-dependent oxidoreductase activity; linked to fatty acid metabolism	-0.834	-0.615
ABCA4	ATP binding cassette subfamily A member 4	Retina-specific ATP-binding cassette (ABC) transporter; associated with retinal diseases	-2.794	-2.913
Discordant				
ASNSP1	Asparagine synthetase pseudogene	Pseudogene	2.776	-5.693
MYH14	Myosin heavy chain 14	Non-muscle myosin involved in cytokinesis, cell motility, and cell polarity; associated with hearing impairment and peripheral neuropathy; predicted ALS gene[4]	-5.120	4.971
NBEAP1	Neurobeachin pseudogene 1	Pseudogene	1.213	-5.991
BMS1P10	BMS1 pseudogene 10	Pseudogene	1.785	-2.262
ENSG00000272234	Novel transcript, antisense to *SEPP1*	lncRNA	2.231	-3.091
CCDC152	Coiled-coil domain containing 152	Protein-coding gene of unknown function	2.421	-4.929
PUS7L	pseudouridine synthase 7 like	Predicted to enable pseudouridine synthase activity; associated with epilepsy	0.333	-0.610
ZSCAN1	Zinc finger and SCAN domain containing 1	DNA-binding transcription factor activity	1.697	-9.783

* ALS, amyotrophic lateral sclerosis; CNS, central nervous system; FC, fold change; lncRNA, long non-coding RNA; PD, Parkinson's disease

** Bolded genes selected for validation by qPCR

*** According to the GeneCards database available at https://www.genecards.org/

Supplementary Table S4. Shared dysregulated miRNAs and their genomic location

miRNA	Genomic coordinate		LogFC p.A382T	LogFC p.G348C
hsa-miR-6832-3p	chr6: 31633787-31633858 [+]	6p21.33	5.39753821	5.09015244
hsa-miR-377-5p	chr14: 101062050-101062118 [+]	14q32.31	-3.4987295	-3.5640906
hsa-miR-432-5p	chr14: 100884483-100884576 [+]	14q32.2	-8.5096634	-7.6425164
hsa-miR-370-3p	chr14: 100911139-100911213 [+]	14q32.31	-6.1090404	-5.9600407
hsa-miR-485-3p	chr14: 101055419-101055491 [+]	14q32.31	-2.7175301	-3.2348732
hsa-miR-487a-3p	chr14: 101052446-101052525 [+]	14q32.31	-2.9450634	-3.1297286
hsa-miR-134-5p	chr14: 101054687-101054759 [+]	14q32.31	-2.7123204	-2.9936839
hsa-miR-495-3p	chr14: 101033755-101033836 [+]	14q32.31	-2.8734665	-2.7943359
hsa-miR-329-3p	chr14: 101026785-101026864 [+]<br>chr14: 101027100-101027183 [+]	14q32.31	-2.7681077	-3.276883
hsa-miR-758-3p	chr14: 101026020-101026107 [+]	14q32.31	-3.1996719	-3.1022029
hsa-miR-487b-3p	chr14: 101046455-101046538 [+]	14q32.31	-3.191169	-3.2420759
hsa-miR-654-5p	chr14: 101040219-101040299 [+]	14q32.31	-2.8213098	-2.9534777
hsa-miR-412-5p	chr14: 101065447-101065537 [+]	14q32.31	-2.9474603	-3.6345566
hsa-miR-656-3p	chr14: 101066724-101066801 [+]	14q32.31	-2.9487844	-4.1468312
hsa-miR-323a-3p	chr14: 101025732-101025817 [+]	14q32.31	-2.6949508	-2.9099091
hsa-miR-376a-3p	chr14: 101040782-101040849 [+]<br>chr14: 101040069-101040148 [+]	14q32.31	-3.4993597	-3.7251577
hsa-miR-409-3p	chr14: 101065300-101065378 [+]	14q32.31	-3.2102507	-3.0777423
hsa-miR-381-3p	chr14: 101045920-101045994 [+]	14q32.31	-3.132852	-3.4426206
hsa-miR-889-3p	chr14: 101047901-101047979 [+]	14q32.31	-3.040022	-3.4464336
hsa-miR-485-5p	chr14: 101055419-101055491 [+]	14q32.31	-2.8677297	-3.1330652
hsa-miR-136-3p	chr14: 100884702-100884783 [+]	14q32.2	-7.2324032	-6.4719432
hsa-miR-411-5p	chr14: 101023325-101023420 [+]	14q32.31	-2.8392333	-3.491725
hsa-miR-433-3p	chr14: 100881886-100881978 [+]	14q32.2	-6.6722867	-6.9097905
hsa-miR-379-3p	chr14: 101022066-101022132 [+]	14q32.31	-3.6183613	-2.809508
hsa-miR-382-5p	chr14: 101054306-101054381 [+]	14q32.31	-2.9233342	-3.2294855
hsa-miR-411-3p	chr14: 101023325-101023420 [+]	14q32.31	-3.1726077	-3.1718531
hsa-miR-493-5p	chr14: 100869060-100869148 [+]	14q32.2	-8.8452075	-6.2919642
hsa-miR-369-5p	chr14: 101065598-101065667 [+]	14q32.31	-3.348575	-3.6018686
hsa-miR-379-5p	chr14: 101022066-101022132 [+]	14q32.31	-2.9522166	-3.1315874
hsa-miR-493-3p	chr14: 101033755-101033836 [+]	14q32.31	-7.4756617	-5.4134923
hsa-miR-1185-2-3p	chr14: 101044198-101044283 [+]	14q32.31	-3.5779433	-3.6520981
hsa-miR-127-3p	chr14: 100882979-100883075 [+]	14q32.2	-6.2099569	-6.7713807
hsa-miR-654-3p	chr14: 101040219-101040299 [+]	14q32.31	-2.9824453	-3.1408796
hsa-miR-655-3p	chr14: 101049550-101049646 [+]	14q32.31	-3.1648455	-3.6508573
hsa-miR-543	chr14: 101031987-101032064 [+]	14q32.31	-2.9567885	-2.6150719
hsa-miR-410-3p	chr14: 101065912-101065991 [+]	14q32.31	-3.0472873	-3.2986066

* FC, fold change

Supplementary Table S5. Predicted TDP-43 binding sites by the RBPmap database[7]

Gene	Genomic coordinate	Motif	Predicted binding sites	Z-score	*P*-value
MIR377	chr14:101062114	guaug	guugaaucacacaaaggcaacuuuu**guuug**	2.214	1.34E-02
MIR370	chr14:100911182	guaug	acgucucugcaguuacacagcucac**gagug**ccugcuggggugggaaccuggucugu	2.051	2.01E-02
	chr14:100911194	guaug	uuacacagcucacgagugccugcug**ggggug**gaaccuggucugucu	2.316	1.03E-02
	chr14:100911206	guaug	cgagugccugcugggguggaaccug**gucug**ucu	2.714	3.32E-03
MIR485	chr14:101055424	gagug	acuug**gagag**aggcuggccgugaugaauucgauuc	2.073	1.91E-02
	chr14:101055426	gagug	acuugga**gagag**gcuggccgugaugaauucgauucau	2.073	1.91E-02
MIR134	chr14:101054689	guaug	ca**gggug**ugugacugguugaccagaggggcau	2.469	6.77E-03
	chr14:101054691	guaug	caggg**ugug**ugacugguugaccagaggggcaugc	3.194	7.02E-04
	chr14:101054693	guaug	cagggu**gugug**acugguugaccagaggggcaugcac	3.194	7.02E-04
	chr14:101054697	guaug	cagggugugu**gacug**guugaccagaggggcaugcacugug	2.551	5.37E-03
	chr14:101054710	gagug	agggugugugacugguugacca**gaggg**gcaugcacuguguucacccuguggg	2.303	1.06E-02
	chr14:101054715	guaug	ggugugugacugguugaccagaggg**gcaug**cacuguguucacccugugggccacc	2.918	1.76E-03
MIR654	chr14:101040221	guaug	gg**guaag**uggaaagauggugggccgcagaaca	1.684	4.61E-02
MIR412	chr14:101065458	guaug	cuggguacgg**ggaug**gauggucgaccaguuggaaaguaau	1.776	3.79E-02
	chr14:101065462	guaug	cuggguacggggau**ggaug**gucgaccaguuggaaaguaauuguu	1.776	3.79E-02
	chr14:101065525	guaug	acuucaccugguccacuagccgucc**guauc**cgcugcag	1.673	4.72E-02
MIR656	chr14:101066758	guaug	guugccugugagguguucacuuucu**auaug**augaauauuauacagucaaccucuu	1.684	4.61E-02
MIR376A2	chr14:101040094	guaug	gguauuuaaaagguagauuuuccuu**cuaug**guuacguguuugaugguuaaucaua	1.929	2.69E-02
MIR889	chr14:101047910	guaug	gugcuuaaa**gaaug**gcuguccguaguauggucucuauau	2.306	1.06E-02
	chr14:101047925	guaug	gugcuuaaagaauggcuguccgua**guaug**gucucuauauuuaugaugauuaaua	3.041	1.18E-03
MIR411	chr14:101023349	guaug	ugguacuuggagagauaguagacc**guaua**gcguacgcuuuaucugugacguaug	2.276	1.14E-02
	chr14:101023356	guaug	uuggagagauaguagaccguauagc**guacg**cuuuaucugugacguauguaacacg	2.276	1.14E-02
	chr14:101023374	guaug	guauagcguacgcuuuaucugugac**guaug**uaacacgguccacuaacccucagua	2.786	2.67E-03
MIR379	chr14:101022087	guaug	agagaugguagacuauggaac**guagg**cguuaugauuucugaccuauguaac	1.816	3.47E-02
	chr14:101022094	guaug	gaugguagacuauggaacguaggcg**uuaug**auuucugaccuauguaacauggucc	1.816	3.47E-02
MIR493	chr14:100869128	guaug	uuugcacauucggugaaggucuacu**gugug**ccaggcccugugccag	1.755	3.96E-02

Supplementary Table S6. Top-scoring predicted compounds by the CMap database[8]

Name*	Description	τ score**		
		p.A382T	p.G348C	Combined
sulpiride	Dopamine receptor antagonist	0.00	1.35	-94.56
tacedinaline (CI-994)	HDAC inhibitor	-1.02	-0.92	-92.30
**XMD-892**	MAP kinase inhibitor	0.00	11.26	-91.09
**QS-11**	ARFGAP inhibitor	-0.49	-14.82	-90.83
bimatoprost	Prostanoid receptor agonist	0.00	0.00	-90.58
**XMD-885**	Leucine rich repeat kinase inhibitor	-40.03	-5.53	-90.49
GR-46611	Serotonin receptor agonist	-1.30	-2.04	-90.17
CS-110266	Dopamine receptor agonist	-1.21	14.28	-89.57
benzohydroxamic-acid	Antifungal	1.80	-2.33	-88.57
**MLN4924 (Pevonedistat)**	Nedd activating enzyme inhibitor	0.39	-49.65	-87.49
**piperlongumine**	Glutathione transferase inhibitor	-71.64	1.06	-87.05
alpha-linolenic-acid	Omega 3 fatty acid stimulant	0.00	1.62	-85.92
4-hydroxy-2-nonenal	Cytotoxic lipid peroxidation product	0.00	0.00	-85.32
**prunetin**	Breast cancer resistance protein inhibitor	-95.71	29.48	-71.45
mitomycin-c	DNA alkylating agent	-93.55	74.75	-69.90
zaldaride	Calmodulin antagonist	0.00	-88.60	0.60

* Bolded compounds selected for further experimental investigation
**Green highlights indicate scores below the score threshold of -85.

Supplementary Data

Supplementary Data are provided as separate files.

Supplementary Data S1. Differentially expressed genes from RNA-seq experiments

Supplementary Data S2. Comparison of observed gene expression changes with other *TARDBP* MN lines and post-mortem ALS spinal cords

Supplementary Data S3. Differentially expressed miRNAs from small RNA-seq experiments

Supplementary Data S4. Predicted targets of dysregulated miRNAs by the miRGate database

Supplementary Data S5. CMap τ scores outputs

Supplementary References

1. Krach, F. *et al.* Transcriptome–pathology correlation identifies interplay between TDP-43 and the expression of its kinase CK1E in sporadic ALS. *Acta Neuropathol.* **136**, 405–423 (2018).

2. Chen, C. X. Q. *et al.* A multistep workflow to evaluate newly generated iPSCs and their ability to generate different cell types. *Methods Protoc.* **4**, 50 (2021).

3. Lépine, S. *et al.* Homozygous ALS-linked mutations in TARDBP/TDP-43 lead to hypoactivity and synaptic abnormalities in human iPSC-derived motor neurons. *iScience* **27**, 109166 (2024).

4. Bean, D. M., Al-Chalabi, A., Dobson, R. J. B. & Iacoangeli, A. A knowledge-based machine learning approach to gene prioritisation in amyotrophic lateral sclerosis. *Genes (Basel).* **11**, 1–17 (2020).

5. Polymenidou, M. *et al.* Long pre-mRNA depletion and RNA missplicing contribute to neuronal vulnerability from loss of TDP-43. *Nat. Neurosci.* **14**, 459–468 (2011).

6. Tollervey, J. R. *et al.* Characterizing the RNA targets and position-dependent splicing regulation by TDP-43. *Nat. Neurosci.* **14**, 452–458 (2011).

7. Paz, I., Kosti, I., Ares, M., Cline, M. & Mandel-Gutfreund, Y. RBPmap: a web server for mapping binding sites of RNA-binding proteins. *Nucleic Acids Res.* **42**, W361-7 (2014).

8. Lamb, J. *et al.* The Connectivity Map: using gene-expression signatures to connect small molecules, genes, and disease. *Science (80-.).* **313**, 1929–1935 (2006).
